# Cockayne syndrome proteins CSA and CSB maintain mitochondrial homeostasis through NAD^+^ signaling

**DOI:** 10.1111/acel.13268

**Published:** 2020-11-09

**Authors:** Mustafa N. Okur, Evandro F. Fang, Elayne M. Fivenson, Vinod Tiwari, Deborah L. Croteau, Vilhelm A. Bohr

**Affiliations:** ^1^ Laboratory of Molecular Gerontology National Institute on Aging National Institutes of Health Baltimore MD USA; ^2^ Department of Clinical Molecular Biology University of Oslo and The Akershus University Hospital Lørenskog Norway; ^3^ Danish Center for Healthy Aging University of Copenhagen Copenhagen N Denmark

**Keywords:** accelerated ageing, aging, AMPK, Cockayne syndrome, mitochondrial maintenance, mitophagy, NAD^+^

## Abstract

Cockayne syndrome (CS) is a rare premature aging disease, most commonly caused by mutations of the genes encoding the CSA or CSB proteins. CS patients display cachectic dwarfism and severe neurological manifestations and have an average life expectancy of 12 years. The CS proteins are involved in transcription and DNA repair, with the latter including transcription‐coupled nucleotide excision repair (TC‐NER). However, there is also evidence for mitochondrial dysfunction in CS, which likely contributes to the severe premature aging phenotype of this disease. While damaged mitochondria and impaired mitophagy were characterized in mice with CSB deficiency, such changes in the CS nematode model and CS patients are not fully known. Our cross‐species transcriptomic analysis in CS postmortem brain tissue, CS mouse, and nematode models shows that mitochondrial dysfunction is indeed a common feature in CS. Restoration of mitochondrial dysfunction through NAD^+^ supplementation significantly improved lifespan and healthspan in the CS nematodes, highlighting mitochondrial dysfunction as a major driver of the aging features of CS. In cerebellar samples from CS patients, we found molecular signatures of dysfunctional mitochondrial dynamics and impaired mitophagy/autophagy. In primary cells depleted for CSA or CSB, this dysfunction can be corrected with supplementation of NAD^+^ precursors. Our study provides support for the interconnection between major causative aging theories, DNA damage accumulation, mitochondrial dysfunction, and compromised mitophagy/autophagy. Together, these three agents contribute to an accelerated aging program that can be averted by cellular NAD^+^ restoration.

## Graphical abstract

Cross‐species analysis in CS postmortem brain tissue, CS cell, mouse, and nematode models indicates that mitochondrial dysfunction is a common feature in CS. Augmenting NAD^+^ levels by supplementing NAD^+^ precursors such as NR (nicotinamide riboside) and NMN (nicotinamide mononucleotide) in CS can ameliorate age‐associated defects and mitochondrial abnormalities.

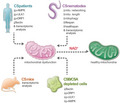

## 1 INTRODUCTION

Patients with Cockayne syndrome (CS) experience cachectic dwarfism and microcephaly, with an average life expectancy of 12 years. CS is considered a segmental aging disease and a model of premature aging. Along with increased genetic damage, mitochondrial dysfunction has been implicated in CS progression; however, the mechanism of this dysfunction needs further exploration (Karikkineth et al., 2017). Cockayne syndrome is predominantly caused by mutations in the genes encoding either the CSA or CSB proteins. CSA (van der Horst et al., 2002) and CSB (van der Horst et al., 1997) are DNA damage response proteins that are involved in transcription‐coupled nucleotide excision repair (TC‐NER) and in nuclear and mitochondrial base excision repair (BER) (Stevnsner et al., 2002). Nucleotide excision repair is a versatile DNA repair pathway as it is responsible for the removal of a wide range of DNA adducts. CSA and CSB are also important for transcription recovery after DNA damage (Mayne & Lehmann, 1982). Various studies have shown that CSB plays a considerable role in transcription and likely is a transcription factor (Epanchintsev et al., 2017; Lake et al., 2014; Okur, et al., 2020).

Mitochondria are highly dynamic organelles that constantly modify their shape, size, and distribution to optimize function and adapt to the energy needs of the cell (Yu & Pekkurnaz, 2018). The decline in mitochondrial function and quality is associated with aging and age‐related diseases and, in particular, neurodegenerative diseases (Johri & Beal, 2012). Similar to other DNA repair‐defective premature aging diseases, such as xeroderma pigmentosum (Fang et al., 2014), ataxia‐telangiectasia (Fang et al., 2016), and Werner syndrome (Fang, et al., 2019), mitochondrial dysfunction is implicated in many CS phenotypes (Scheibye‐Knudsen et al., 2012, 2014). The mechanisms of mitochondrial dysfunction underlying CS remain unclear but may involve the impairment of the NAD^+^ (nicotinamide adenine dinucleotide, oxidized)–mitophagy axis (Fang et al., 2014, 2016, 2019; Scheibye‐Knudsen et al., 2012, 2014). NAD^+^ is a fundamental molecule in cellular energy metabolism and signaling pathways and is essential for adaptive responses of cells to bioenergetics and oxidative stress. There is an age‐dependent depletion of cellular NAD^+^, which may be a major driver of both pathological and biological aging (Gilmour et al., 2020). Many molecular mechanisms are involved in the multi‐faceted effects of NAD^+^ on longevity and healthspan, including the induction of mitophagy, a cellular self‐recognition, engulfment, degradation, and recycling of damaged and superfluous mitochondria (Gilmour et al., 2020). In CS, the accumulation of unrepaired DNA damage may lead to mitochondrial dysfunction through increased PARP‐1‐mediated NAD^+^ consumption (Fang et al., 2014; Scheibye‐Knudsen et al., 2014). Decreased NAD^+^ levels lead to decreased activity of SIRT‐1, an NAD^+^‐dependent deacetylase that maintains mitochondrial homeostasis through coordination of elimination of damaged mitochondria via mitophagy and mitochondrial biogenesis via activation of PGC‐1α and FOXOs (Fang et al., 2014). This phenomenon has also been observed in other DNA repair disorders (Fang et al., 2014, 2016). Notably, rapamycin treatment or dietary restriction, which up‐regulate mitophagy, has shown promise in CS models (Scheibye‐Knudsen et al., 2012). It is therefore of interest whether increasing NAD^+^ levels through NAD^+^ precursor supplementation can ameliorate symptoms of mitochondrial dysfunction in CS.

Our cross‐species and unbiased transcriptomic analysis on CS postmortem brain tissue, CS mouse cerebellum, and CS *Caenorhabditis elegans* (*C*.* elegans*) models showed that Gene Ontology (GO) terms associated with mitochondria are changed in all CS models and are normalized with NAD^+^ supplementation in worms and mouse models of CS. We also report that various mitochondrial abnormalities such as increased mitochondrial length and reduced mitochondrial networking are present in CS worms and are restored by NAD^+^ precursor treatment. Lastly, the activity of several key proteins of mitochondrial homeostasis such as AMPK and ULK‐1 was impaired in the CS postmortem brain tissues. Cellular and tissue NAD^+^ supplementation restored the activity of these proteins in primary cell lines deficient in CSA or CSB. This has implications for intervention in CS and possibly aging.

## 2 RESULTS

### 2.1 Microarray analysis of CS patient cerebellum samples

**Figure 1:**
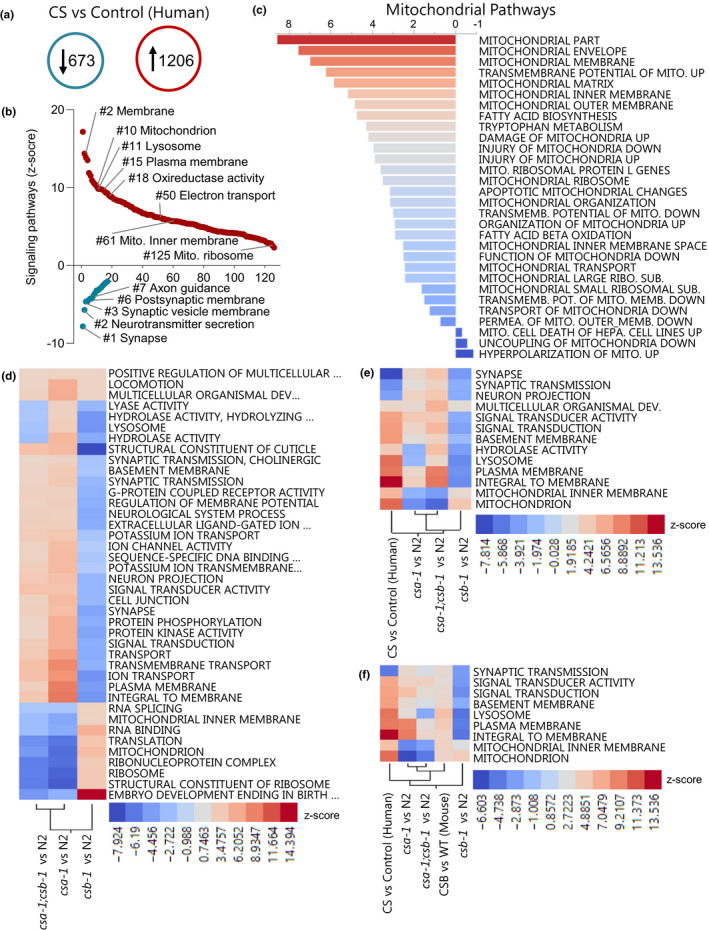
The transcriptomic analysis in CS postmortem brain tissue, CS mouse, and *Caenorhabditis elegans* models. (a) The number of up‐ and down‐regulated genes (fold change≥1.5 and *p*‐value≤0.05) in CS patient cerebellum samples compared to healthy controls. (b) GO term Z‐scores comparing human CS and control patients. (c) The alteration in mitochondrial pathways (Z‐scores) in human CS vs control patients. (d) Heatmaps of GO term Z‐scores showing changes between CS worms compared to N2 worms. Hierarchical clustering showing (e) various GO terms commonly affected between human CS patient and CS worms and (d) GO terms that are commonly affected in CS mouse and worm models, and CS brain samples. A *p*‐value ≤0.05 and an absolute value Z‐score of 2.0 were the cutoffs used for significance.

The loss of CSA and CSB proteins compromise the central nervous system function, with patients exhibiting varying degrees of demyelination, neuronal loss, calcification, gliosis, and cerebellar atrophy (Karikkineth et al., 2017). To examine changes in gene expression in the cerebellum of CS patients, microarray analysis was conducted using frozen cerebellar samples from CS patients and age‐matched controls from the NIH NeuroBioBank's Brain and Tissue repository at the University of Maryland, Baltimore (Table S1). Since the origin of the gene mutations in these patients is unknown, hierarchical clustering was performed on the samples to see whether the CS patient‐derived samples clustered together (Figure S1 and S2a). From this analysis, two samples, CS2 and CS4R, clustered more closely with the controls than the CS patients and were therefore removed from further array analysis. We identified 1,206 up‐regulated and 673 down‐regulated genes in CS compared to non‐CS controls (Figure 1a). Gene expression analysis on the cerebellum of CS patients was previously performed by another group (Wang et al., 2014). We compared the gene expression patterns in the two independent microarrays and found that there is an almost identical match of up‐ and down‐ regulation of the common genes (Figure S2b). These results suggest that the gene expression changes in our array are accurate and CS‐dependent. We next used gene set enrichment analysis to parse the genes into various GO terms and identified 126 (88%) up‐regulated and 17 (12%) down‐regulated terms (Figure 1b and Table S2). Notably, all of the terms associated with synapse and neuronal function were significantly down‐regulated (Figure 1b) in CS. In contrast, many terms associated with mitochondrial function, immune response, and stress response pathways were up‐regulated (Figure 1b and Figure S3a). Taken together, the signature of changes in CS patient samples relative to controls suggests a proinflammatory, oxidative stress environment with neuronal cells suffering from mitochondrial and synaptic dysregulation. Given that many mitochondrial GO terms were altered in CS patients, we next specifically focused on mitochondrial dysfunction and generated a map of affected mitochondrial pathways in CS (Figure 1c). We detected up‐regulation of key mitochondrial pathways that control mitochondrial structure and biogenesis such as mitochondrial ribosome, membrane envelope, and matrix. Interestingly, the pathways that utilize NAD^+^ or are involved in NAD^+^ biosynthesis such as tryptophan metabolism, fatty acid biosynthesis, and fatty acid beta‐oxidation were up‐regulated in CS brain samples. Previously, we implicated oxidative stress, persistent DNA damage signaling, and NAD^+^ depletion as contributors to mitochondrial dysfunction in mice with CSB deficiency (Scheibye‐Knudsen et al., 2012, 2014). Our microarray data of human postmortem brain tissues show cross‐species consistency of these affected terms/pathways and are therefore of great interest in understanding CS pathology.

### 2.2 Microarray analysis of Cockayne syndrome worm models

Transcription‐coupled nucleotide excision repair is important for the DNA repair pathway in *C*. *elegans* (Lans & Vermeulen, 2011), and mutations in both *csa*‐*1* and *csb*‐*1* lead to increased UV sensitivity, suggesting that the function of these proteins is conserved in *C*.* elegans* (Babu et al., 2014; Bianco & Schumacher, 2018). We sought to evaluate *csa*‐*1(tm4539)* and *csb*‐*1(ok2335)* worm mutants to understand the shared or unique features of the disorder. Gene expression microarrays were performed on day 7 adult worms and compared to N2 worms to determine significantly changed GO terms and how these compare to those observed in CS patients. After the selection of terms with *p*‐values lower than 0.05 and an absolute value pathway Z‐score cutoff of 2.0, the remaining 40 GO terms and their respective Z‐scores were sorted by clustering in Figure 1d. Interestingly, *csa*‐*1(tm4539)* and *csb*‐*1(ok2335)* worms had opposite pathway changes for a majority of the terms. Throughout the dataset, *csa*‐*1(tm4539)*;*csb*‐*1(ok2335)* worms partitioned more with *csa*‐*1(tm4539)* than *csb*‐*1(ok2335)* worms, suggesting that *csa*‐*1* drives the double mutant phenotype. However, there was a shortlist of terms, including lysosome and hydrolase activity, where the *csa*‐*1(tm4539)*;*csb*‐*1(ok2335)* worms segregated with *csb*‐*1(ok2335)* worms. Previously, we reported that CS mice have an accumulation of dysfunctional mitochondria, with ribosomal and mitophagy defects (Scheibye‐Knudsen et al., 2012). These types of GO terms were significantly changed in the nematodes with mitochondrial and ribosomal pathways up‐regulated in *csa*‐*1(tm4539)* and *csa*‐*1(tm4539)*;*csb*‐*1(ok2335)*, but down‐regulated in *csb*‐*1(ok2335)*. The lysosomal pathway, which is important for mitophagy (Raimundo et al., 2016), showed an opposite trend with up‐regulation in *csa*‐*1(tm4539)* but down‐regulation in *csb*‐*1(ok2335)* and *csa*‐*1(tm4539)*;*csb*‐*1(ok2335)* worms. The presence of changes in these GO terms, despite their direction, suggests the dysregulation in mitochondrial and ribosomal pathways in CS worms. Notably, we also observed that certain GO terms such as “Response to reactive oxygen species” or “Galactosyltransferase activity” were significantly changed only in *csa*‐1;csb‐1 double mutant strain but not in single mutants, suggesting pathways that are synergistically impacted by CSA and CSB (Figure S3b).

To understand the similar pathology in all CS models, we compared the GO terms in human CS patients and CS worms. Only 13 GO terms, which were associated with mitochondria, synapse, membrane, and signal transduction, were in common (Figure 1e). Interestingly, hierarchical clustering showed that *csb*‐*1(ok2335)* worms cluster more closely to human CS than *csa*‐*1(tm4539)* or *csa*‐*1(tm4539)*;*csb*‐*1(ok2335)* worms when the largest group of human CS terms listed in Figure S3a were compared (Figure S3c). To understand the similar pathology in all CS models, we also included GO terms from the cerebellum of CSB mutant mice and their controls from a previously published study (Scheibye‐Knudsen et al., 2014). CSB mice recapitulate the features of small body size, hearing loss, and retinal degeneration (Gorgels et al., 2007; Okur, et al., 2020). In addition to these features, we showed the presence of reduced muscle strength in old CSB mutant mice (Figure S4). We found that the terms associated with synaptic transmission, cellular signaling, membrane formation, lysosome, and mitochondria were the only GO terms that are commonly affected in all CS models and CS brain samples (Figure 1f).

**Figure 2:**
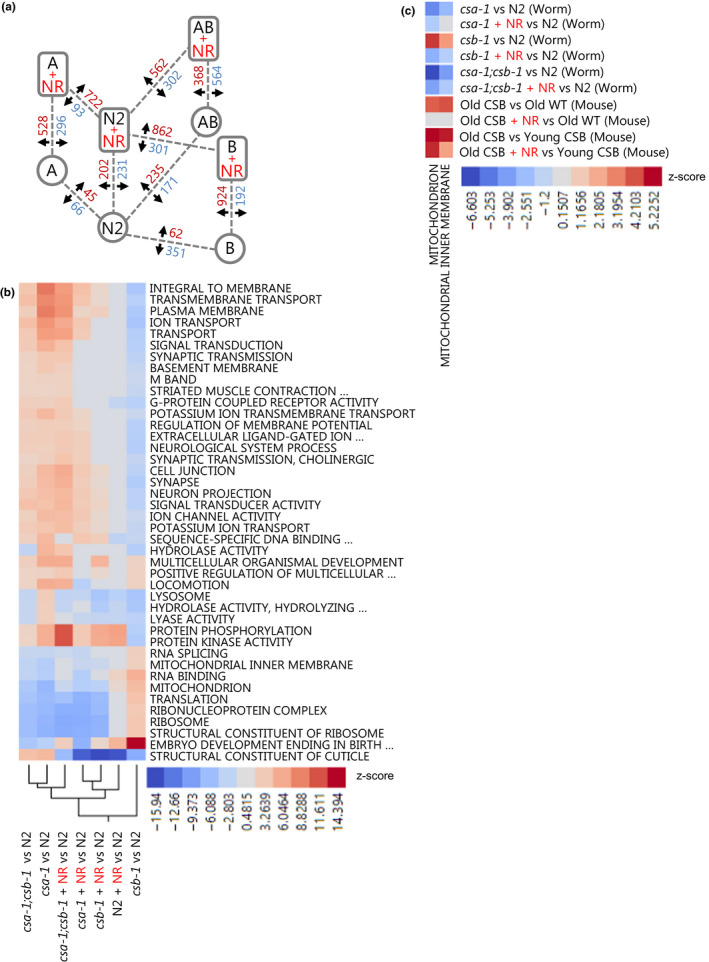
Transcriptomic analyses from CS *Caenorhabditis elegans* models and CS mouse without/with NR treatment. (a) The up‐ and down‐regulated genes (fold change ≥1.5 and *p*‐value ≤0.05) in CS strains vs. N2 with or without NR treatment. (b) Heatmap showing Z‐score changes of the various GO terms from CS worms ±NR treatment. (c) Heatmap showing Z‐score changes of the GO terms related to mitochondria and mitochondrial inner membrane from CS worm and mouse models ±NR treatment. Terms were considered significant if they had *p*‐values lower than 0.05 and an absolute value pathway Z‐score 2.0.

We previously showed that NAD^+^ supplementation with NR was of benefit in DNA repair‐deficient mouse and worm models (Fang et al., 2014, 2016). Thus, we sought to determine whether NR could also modulate gene expression patterns and GO terms in this system. Microarray analysis was done on adult day 7 worms that were treated with water (vehicle, veh) or NR beginning the late L4 stage. Each genotype was compared to N2 vehicle worms for consistency. Overall, NR treatment modulated the gene expression profile in all CS worm strains (Figure 2a) and caused CS mutant nematodes to cluster more closely with N2 (Figure 2b). To assess the pathways that are specially targeted by NAD^+^ supplementation, we examined the Z‐score of individual GO terms that were altered in CS models but restored with NR treatment. The terms mitochondrion and mitochondrial inner membrane were the only common GO terms in CS mice and nematodes with Z‐score values that were normalized after NR treatment (Figure 2c and Figure S5). Altogether, our comprehensive transcriptomic analysis suggests that the alterations in mitochondria play a central role in CS pathology and are conserved across animal models of CS.

### 2.3 NAD^+^ replenishment in CS nematodes

**Figure 3:**
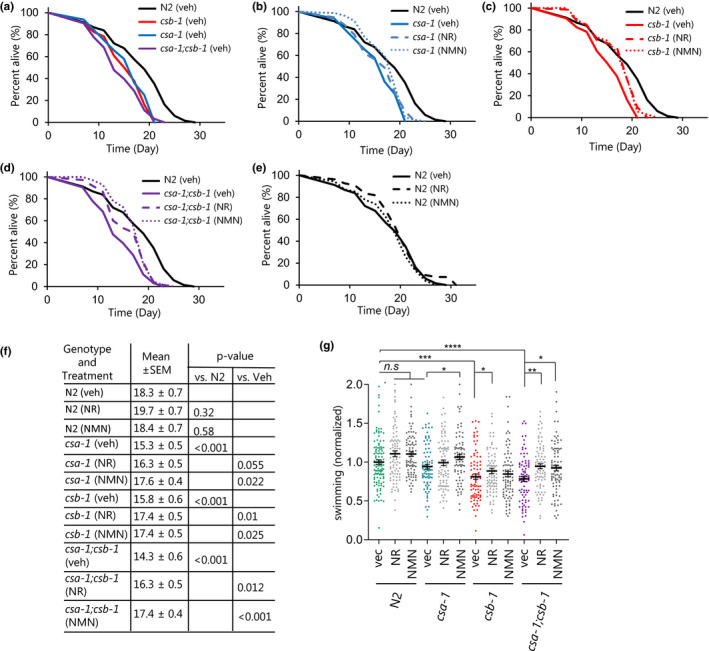
NAD^+^ replenishment rescues lifespan and healthspan of CS mutant *C*.* elegans*. (a‐e) Effects of NAD^+^ supplementation on the lifespan of N2, *csa*‐*1(tm4539)*, *csb*‐*1(ok2335)*, and *csa*‐*1(tm4539)*; *csb*‐*1(ok2335) worms* at 25°C with or without 1 mM NR and NMN treatment. A representative set of data from two biological repeats (The raw data is available in Data S1, which also includes an additional independent biological experiment testing N2 and *csa*‐*1* life span without/with 1 mM NR and NMN treatment). The log‐rank test was used, with *p* ≤ 0.05 was considered significant. (f) The tabular representation of lifespan data. (g) Effect of NR/NMN on swimming in N2 and CS worms at D8. Data are represented as mean ±SEM (Total of 80–100 worms for each condition combined from three biologically independent experiments; Two‐way ANOVA with Tukey's post hoc test was used to determine significant differences. *p ≤ 0.05, **p ≤ 0.01, ***p ≤ 0.001, ****p ≤ 0.0001 and n.s., not significant.).

The efficacy of NAD+supplementation in improving mitochondrial health was previously reported (Fang et al., 2014, 2016). We thus wondered whether targeting the pathological mitochondrial phenotype via NAD^+^ supplementation can alleviate disease phenotypes. To address this, *csa*‐*1(tm4539)*, *csb*‐*1*, and *csa*‐*1(tm4539)*;*csb*‐*1* worms were analyzed for mean and maximum lifespan following NAD^+^ supplementation using two NAD^+^ precursors, NR (1 mM) or NMN (1 mM), from L4 stage (Figure 3a‐f and Figure S6a). In the absence of drug treatment, N2 worms lived for 18.3 ± 0.7 days, while all the mutants showed reduced lifespans (Figure 3a,f). *csa*‐*1(tm4539)* showed a 16% reduction (15.3 ± 0.5 days), *csb*‐*1(ok2335)* a 13.6% reduction (15.8 ± 0.6 days), and *csa*‐*1(tm4539)*;*csb*‐*1(ok2335)* a 22% reduction (14.3 ± 0.06 days) (Figure 3a). After drug treatments, in comparison with vehicle controls, both NAD^+^ supplement‐treatment groups showed significantly improved lifespan of each genotype (Figure 3b‐d,f). No significant benefit of either drug was observed in N2 worms (Figure 3e‐f).

We next assessed healthspan and found that swimming motions were reduced in all CS genotypes except the *csa*‐*1(tm4539)* strain which displayed a slight reduction in swimming, but it did not reach significance (Figure 3h). Notably, NR or NMN treatment significantly improved swimming at day (D) 8 in all CS genotypes (Figure 3h). The pumping rate or maximum velocity was similar across all genotypes at the ages of 6 and 9 days (Figure S6b‐c). Collectively, the worm data suggest that NAD^+^ augmentation extends lifespan and improves some of the healthspan parameters in the *csb*‐*1(ok2335)*, and *csa*‐*1(tm4539)*;*csb*‐*1(ok2335)* worms.

### 2.4 Mitochondrial assessment in CS nematodes

**Figure 4:**
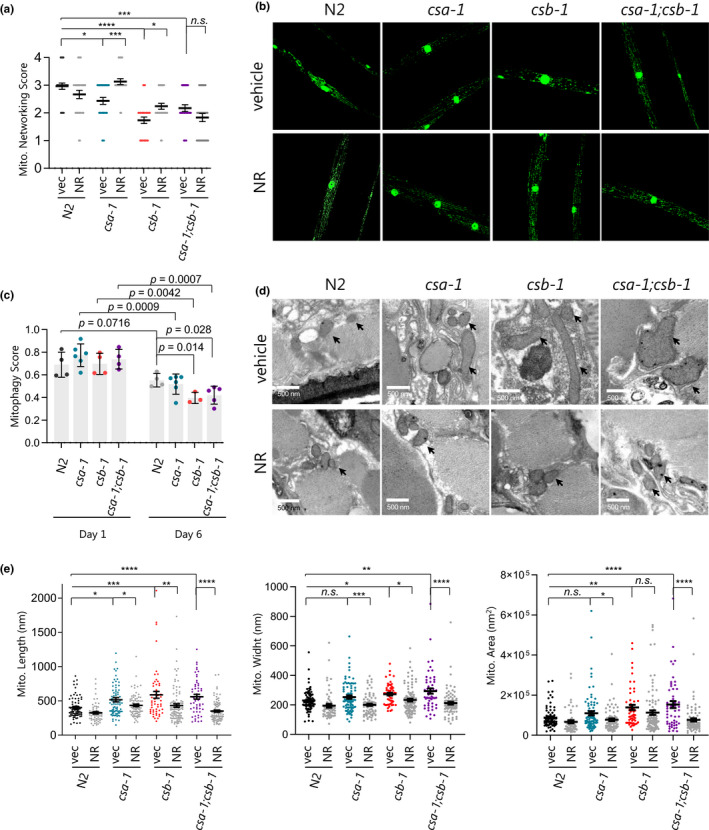
CS worms have reduced mitochondrial networking and mitophagy. (a,b) The *myo*‐*3*::*gfp* reporter was expressed in both the nucleus and mitochondria to mark non‐pharyngeal body wall muscle cells in the worms. Representative images and quantified scores of muscle mitochondrial morphology of adult D7 N2 and CS worms. Data are shown as mean ±SEM (Total of 29‐30 images of muscle cells from each group of worms (N = 10), Two‐way ANOVA with Tukey's post hoc test was used. *p ≤ 0.05, **p ≤ 0.01, ***p ≤ 0.001, ****p ≤ 0.0001 and n.s., not significant.). (c) The mitophagy reporter strain *N2*;*Ex(pmyo*‐*3∷dsred∷lgg*‐*1*;*pdct*‐*1∷dct*‐*1∷gfp)* was crossed with *csa*‐*1(tm4539)*, *csb*‐*1(ok2335)* or *csa*‐*1(tm4539)*; *csb*‐*1(ok2335) worms* and imaged for muscle cells at D1 and D6 to measure the colocalization coefficient between DSRED∷DCT1 and LGG1∷GFP. Data are shown in mean ±SEM (n = 3‐6 worms; two‐tailed *t* test). (d) Mitochondrial structure changes were evaluated via electron microscopy (arrows indicate mitochondria). (e) Mitochondrial length, width, and area were quantified. Data are shown in mean ±SEM (n = 16–19 worms for each condition; Two‐way ANOVA with Tukey's post hoc test was used. *p ≤ 0.05, **p ≤ 0.01, ***p ≤ 0.001, ****p ≤ 0.0001 and n.s., not significant.)

We previously reported an increased oxygen consumption rate in *C*. *elegans* worms defective in *csa*‐*1(tm4539)* and *csb*‐*1(ok2335)* (Scheibye‐Knudsen et al., 2016), implicating mitochondrial dysfunction in CS worms. Indeed, we found that the GO term mitochondrion was one of the most significantly changed terms when comparing CS to N2 worms (Figures 1 and 2). Along with the mitochondrial changes, the GO term for the lysosome was significantly changed. Mitophagy is a regulated process that involves the degradation of damaged mitochondria by lysosomes. This process plays an important role in mitochondrial homeostasis, neuroprotection, and even healthy longevity in laboratory animal models (Fang, et al., 2019). While we have characterized mitophagy in the CSB^m/m^ mice (Scheibye‐Knudsen et al., 2012), the changes of mitochondrial morphology and mitophagy in the worm models with CSA mutation or CSA/CSB double mutations are not known. We therefore sought to characterize mitochondrial networking and mitophagy in the CS *C*.* elegans* strains. In order to visualize the mitochondrial networking in the *C*.* elegans* body wall muscle tissue, we crossed *csa*‐*1(tm4539*), *csb*‐*1(ok2335)*, and *csa*‐*1(tm4539)*;*csb*‐*1(ok2335)* mutants to a *myo3*::*gfp* reporter strain (N2 background) in which GFP is targeted to the mitochondria and the nuclei of body wall muscle cells. For these experiments, late L4 stage worms were treated with 1 mM NR. At day 7, worms were immobilized using Levamisole (100 mM) and imaged under a confocal microscope. All CS worm mutants exhibited significantly diminished levels of mitochondrial networking relative to N2 veh (Figure 4a‐b and Figure S7), and this phenotype was rescued with NR treatment in *csa*‐*1(tm4539)* and *csb*‐*1(ok2335)* strains. To directly visualize mitophagy in worms, the strains were crossed with DCT‐1::GFP and LGG‐1::RFP expressing worms (Palikaras et al., 2015). Colocalization of the two markers is indicative of mitophagy, and from this, we can derive a “mitophagy score.” On day 6, *csb*‐*1(ok2335)* and *csa*‐*1(tm4539)*;*csb*‐*1(ok2335)* worms displayed reduced mitophagy compared to N2 worms, by 28 and 23 percent, respectively, while there were no significant differences on day 1 (Figure 4c and Figure S8). Notably, all CS genotypes exhibited a progressive reduction of mitophagy upon aging compared to Day 1, while there was only a trend toward a reduction in the N2 worms (Figure 4c, compare mitophagy score values on Day 1 to Day 6). To gain more insight into mitochondrial health in CS, we next assessed mitochondrial morphology in the CS strains by electron microscopy (Figure 4d‐e). Consistent with defective mitophagy in the CS worms, we observed increased mitochondrial length in all CS genotypes, which was restored with NR treatment. Notably, we found that NAD^+^ supplementation also restored increased mitochondrial width in *csb*‐*1(ok2335) and csa*‐*1(tm4539)*;*csb*‐*1(ok2335)* strains and mitochondrial area in *csa*‐*1(tm4539) and csa*‐*1(tm4539)*;*csb*‐*1(ok2335)* strains.

### 2.5 Mitochondrial dynamics in CS patient cerebellum samples

**Figure 5:**
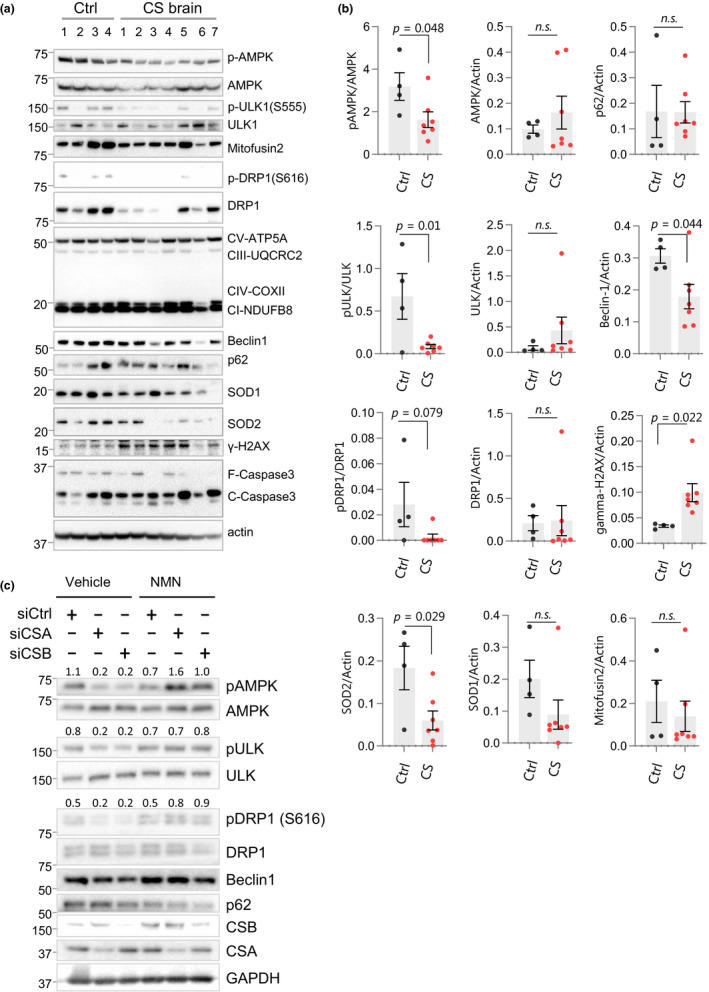
Protein expression levels in CS patient cerebellum samples. (a) Western blots showing changes in the expression of designated proteins from control and CS patient cerebellum samples. The characteristics of the patient material are shown in Table S3. (b) Quantitation of western blot data in *(a)*. Data are shown in mean ±SEM (n = 3–6 worms; Two‐tailed *t* test.). (c) NAD^+^ supplementation rescues AMPK signaling in CSA and CSB‐depleted fibroblasts. Western blot data showing changes in the expression of designated proteins from control and CSA‐ or CSB‐depleted primary fibroblast cell line (passage 9). Blots shown are representative of three independent biological repeats. The values above the blots show the densitometry quantification of the relative phosphorylation levels of the indicated proteins.

The presence of impaired mitochondrial networking and morphology in CS worms (Figure 4) suggests a dysregulation in mitochondrial homeostasis. We therefore assessed CS patient cerebellum samples for the activity of the proteins that have a key role in the turnover or dynamics of mitochondria (e.g. AMPK, DRP‐1, and ULK1). AMPK directly phosphorylates downstream targets like PGC1α, which is important for mitochondrial biogenesis, and ULK1 (autophagy protein unc‐51 like autophagy activating kinase 1) is critical for mitophagy and promotes mitochondrial homeostasis. We consistently observed a reduction in the activation of AMPK and its downstream target, ULK1, in CS patient samples (Figure 5a,b). Further, the levels of Beclin1 were also decreased, suggesting a defect in the initiation of cellular autophagy in CS. In line with the findings of enlarged mitochondria in CS nematodes (Figure 4d‐e), we observed a trend toward a reduction in pDRP1 activity, a protein important for mitochondrial fission, in CS brain samples (Figure 5 and Figure S9). As previously reported (Pascucci et al., 2018), the levels of Gamma‐H2AX, a marker of DNA damage, were increased in CS. The oxidative stress pathway was up‐regulated in the microarray, so we also measured levels of the reactive oxygen detoxifying enzymes superoxide dismutase 1 (SOD1, cytoplasmic) and 2 (SOD2, mitochondrial). Although there was no significant change in SOD1 levels, we found that SOD2 levels increased in CS.

### 2.6 Restoration of AMPK signaling in CSA‐ and CSB‐depleted fibroblasts with NAD^+^ supplementation

The effect of AMPK on NAD^+^ metabolism was previously published (Canto et al., 2009). To investigate whether NAD^+^ supplementation could modulate the expression and activation of mitophagy and autophagy‐related proteins, we treated primary fibroblasts with NMN following CSA or CSB impairment. In line with the findings in CS brain samples, we observed lower levels of activated AMPK (p‐AMPK), pULK1 and pDRP1 in CSA and CSB‐deficient primary fibroblasts (Figure 5c and Figure S10). Notably, NAD^+^ supplementation restored phosphorylation of these proteins, which is predicted to increase their activity (Figure 5c). Beclin1 levels were slightly lower in CSA and CSB‐depleted cells compared to control cells but increased significantly following NMN treatment. The p62 protein levels in CSA and CSB‐deficient cells show no difference from the control group but decrease following NMN treatment in all samples, suggesting that cellular autophagy is increased after NAD^+^ supplementation. Collectively, these findings suggest that impaired activation of AMPK and ULK1 along with DRP1 leads to dysregulation of mitochondrial fission and degradation and causes subsequent defects in mitochondrial homeostasis. NAD^+^ supplementation restores these features and improves mitochondrial quality in CS.

## 3 DISCUSSION

Mitochondrial homeostasis involves coordination between mitochondrial biogenesis and mitophagy. If these processes become uncoordinated, mitochondrial dysregulation can ensue. Hence, mitochondrial dysfunction is causative in many human ailments, including neurological abnormalities (Johri & Beal, 2012). It is important to investigate common phenotypes between human and animal models of CS to explore conserved pathways. Here, we report that mitochondrial dysfunction is a common feature across CS patients and animal models. Comparative transcriptomic analysis of CS patient cerebellum samples and CS animal models like *C. elegans* and mice converge to implicate mitochondrial dysfunction in CS. This is in line with the dysfunctional mitochondrial dynamics such as increased mitochondrial membrane potential, superoxide production, and increased oxygen consumption rates that are commonly seen in many CS models (Scheibye‐Knudsen et al., 2012, 2014). Notably, we observed a stronger impact of csb‐1(ok2335) on mitochondrial morphology, networking, and mitophagy than the csa‐1(tm4539) mutant has (Figure 4). This was also reflected in mitochondria‐associated swimming behavior (Figure 3g). Interestingly, our transcriptomic analysis also revealed that csb‐1(ok2335) worms cluster more closely to human CS than csa‐1(tm4539) worms on the common mitochondria‐associated GO terms (Figure S3c and S5).

Notably, unlike the previous report, we observed a shorter lifespan of *csb*‐*1(ok2335)* worms potentially due to different experimental settings leading to temperature‐induced stress and exacerbation of the DNA repair deficiency on lifespan (Lans et al., 2013). Nevertheless, we observed that NAD^+^ augmentation improves lifespan in CS worm models. Also, NAD^+^ supplementation enhanced mitochondrial networking and led to the normalization of mitochondrial morphology via restoration of mitochondrial length. Specifically, mitochondrion and mitochondrial inner membrane GO terms were the only GO terms that NR treatment nearly reverted to wild type in both CS mouse and worm models, suggesting NR's therapeutic effects are mediated significantly through mitochondrial homeostasis in CS. Interestingly, unlike its effect on single mutants, NAD+augmentation failed to improve mitochondrial networking in the *csa*‐*1*;*csb*‐*1* double mutant (Figure 4a). This apparently inconsistent result may be due to the synergistic impact of CSA and CSB on an NAD^+^‐independent pathway regulating mitochondrial networking. Indeed, our transcriptomic analysis in CS worms identified GO terms that are only altered with *csa*‐*1 and csb*‐*1* double mutation but not significantly affected by single mutation or NR treatment (Figure S3b). Investigating these CSA‐ and CSB‐mediated pathways might provide novel insights into CS disease mechanism, particularly in the context of mitochondrial homeostasis.

**Figure 6:**
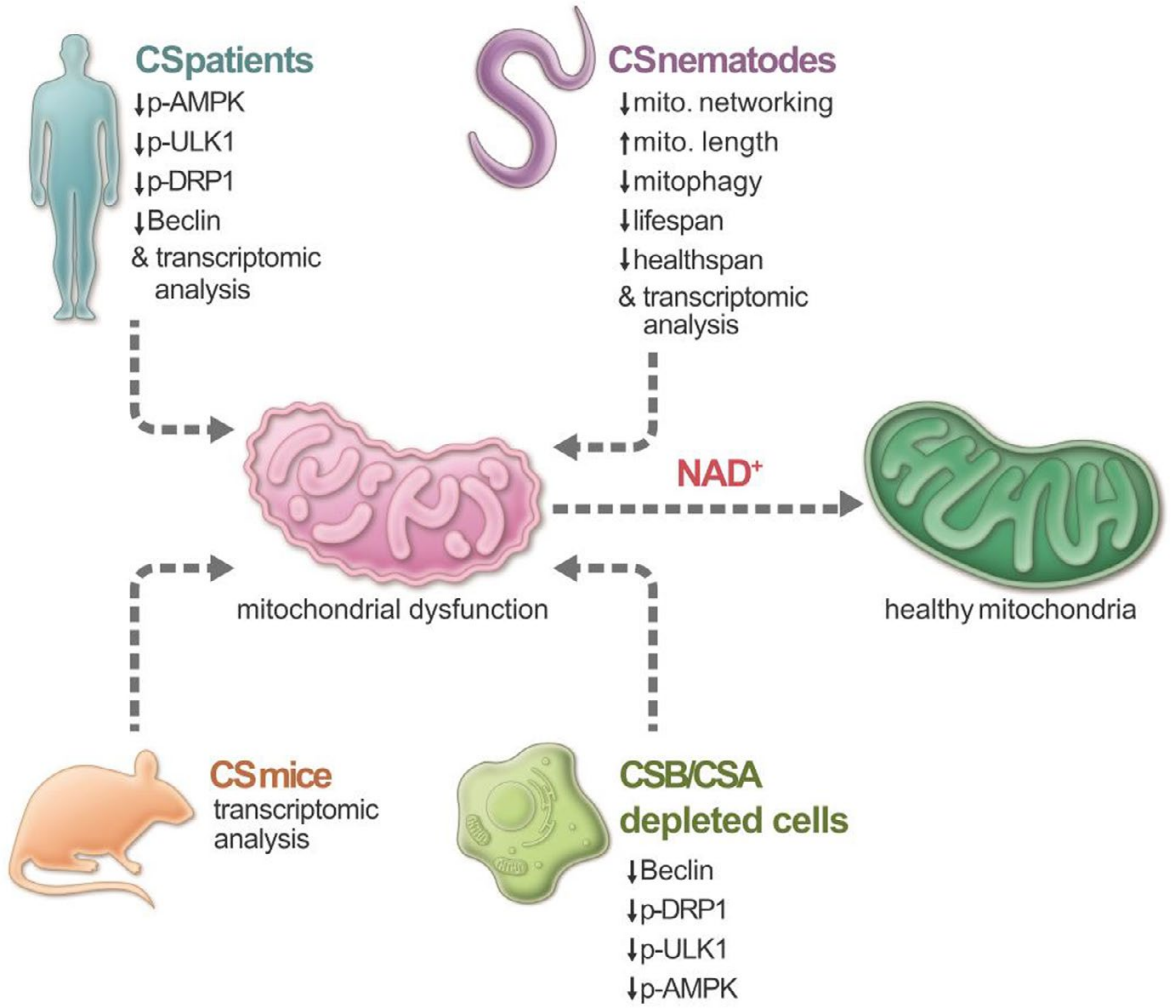
We propose CS mutation leads to cellular NAD^+^ reduction that impairs mitochondrial homeostasis through down‐regulation of the activities of p‐AMPK and p‐ULK1. NAD^+^ levels decline during the aging process, resulting in many age‐associated pathologies like mitochondrial dysfunction. Restoring NAD^+^ level by supplementing NAD^+^ precursors such as NR (nicotinamide riboside) and NMN (nicotinamide mononucleotide) can ameliorate these age‐associated defects.

Our data suggest that impairment of neuronal pathways along with mitochondrial dysregulation contributes to the neurodegeneration in CS. CSB has been known to localize to mitochondria and participate in DNA repair and transcription (Aamann et al., 2010; Berquist et al., 2012). Neurological degeneration is observed in many conditions with DNA repair deficiency including CS (Karikkineth et al., 2017). Cells with compromised mitophagy contribute to neuronal death and thus cause neurodegeneration (Fang, et al., 2019). Our recent report suggests that NAD^+^‐dependent SIRT1 activity regulates mitophagy through a DAF‐16‐DCT1 pathway (Fang et al., 2016). AMPK is associated with NAD^+^ metabolism and SIRT1 activity, and its activation regulates ULK1/DRP1 proteins necessary for mitophagy (Egan et al., 2011). Protein expression from the brain samples of CS patients showed dysregulation of AMPK and decreased phosphorylation of its downstream target, ULK1, which is necessary for ULK1's translocation to damaged mitochondria for mitophagy. Although we did not observe a significant reduction in mitofusin 2, there was a trend toward reduction in phosphorylated dynamin‐like protein DRP1, which mediates the mitochondrial fission and subsequent fragmentation to undergo mitophagy (Wang & Youle, 2016). This finding is in line with the increased mitochondrial length in all CS worm strains, suggesting defects in mitochondrial fragmentation. AMPK helps in recruiting DRP1 to the mitochondrial outer membrane and thus regulates the morphology of mitochondria (Li & Chen, 2019; Toyama et al., 2016). This imbalanced regulation of mitophagy was further supported by the increased levels of DNA damage and ROS (Andrade et al., 2012). Interestingly, we noted that NMN treatment helps regulate AMPK pathway proteins. Thus in CS, increased DNA damage causes increased PARP‐1‐mediated NAD^+^ consumption, thereby leading to depletion in the NAD^+^ pool (Scheibye‐Knudsen et al., 2014). This NAD^+^ pool is regulated by the AMPK‐ULK1/DRP1 pathway, and the dysregulation of CS proteins generates a mitochondrial dysfunction phenotype in CS (Figure 6). The extracellular NAD^+^ degradation produces increased adenylate levels in the cell and increases the intracellular ATP levels by activating AMPK through modulation of the ATP/AMP ratio (Zhang et al., 2018). AMPK senses stress to initiate mitochondrial fission to eliminate damaged mitochondria while at the same time inducing mitochondrial biogenesis to regulate mitochondrial homeostasis (Toyama et al., 2016). Given the central role of NAD^+^ metabolism and mitochondria in energy production, CSA and CSB play an important role to modulate the AMPK‐ULK1/DRP1 pathway to maintain mitochondrial homeostasis.

In closing, these results illustrate the underlying molecular mechanisms of CS proteins (CSA and CSB) in preventing premature age‐induced mitochondrial dysfunction. Moreover, the interplay between NAD^+^ supplementation, mitochondria, and mitophagy provides insight into the development of therapeutic strategies in the treatment of DNA repair‐accelerated aging models like CS.

## 4 METHODS

### 4.1 *Caenorhabditis elegans* strains and cultivation

All *C*.* elegans* strains were maintained and grown on standard nematode growth medium (NGM) as previously described (Brenner, 1974). Bristol strain N2 (wild type) and *myo3*::*gfp* reporter strains were obtained from the Caenorhabditis Genetics Center (CGC). Worm populations were synchronized via egg lay, and all experiments were conducted at 25°C unless otherwise noted. *csa*‐*1(tm4539)* and *csb*‐*1(ok2335)* were provided by Dr. Bjorn Schumacher (University of Cologne, Cologne, Germany). The strains were backcrossed to N2 multiple times to clear out unknown mutations. Furthermore, the worms were thawed from the original stocks regularly to avoid maintaining for extended generations. The mitophagy reporter strain *N2*;*Ex(pmyo*‐*3∷dsred∷lgg*‐*1*;*pdct*‐*1∷dct*‐*1∷gfp)* was provided by Dr. Nektarios Tavernarakis (Institute of Molecular Biology and Biotechnology, Crete, Greece) and described previously (Palikaras et al., 2015).

### 4.2 *Caenorhabditis elegans* drug treatment

Worms were treated with 100 µM fluorodeoxyuridine (FUDR) in order to prevent egg‐laying throughout the study (unless otherwise noted), as described previously (Mitchell et al., 1979). Worms were transferred manually to plates containing FUDR at the late L4 developmental stage. Worms were treated with NAD^+^ precursors nicotinamide riboside (NR) and nicotinamide mononucleotide (NMN) at a final concentration of 1 mM in water. Drug plates changed every three days. Water was used as vehicle control. The drug was added directly to seeded plates one day ahead of the experiments (unless otherwise noted). Worms were then transferred to the treatment plates by hand.

### 4.3 *Caenorhabditis elegans* lifespan analysis

Lifespan analysis was conducted as described previously (Fang et al., 2014) with minor modifications. Experiments were conducted at 25°C unless otherwise noted. Briefly, worm populations were synchronized via egg lay and randomly selected (to avoid selecting bigger and more active worms) and transferred to treatment plates at the L4 stage (48 h post egg lay at 25°C). Each group contained 3 plates. 35–40 worms were plated in each of the 6.5‐cm petri dish with NGM and *Escherichia coli* (*E. coli*, L4440) seeded. Drugs (NR) were added to the NGM plates with *E*.* coli* 12 h ahead to enable equally NR spreading in the agar at the time of experiment. Animals were transferred every two days to freshly prepared NGM plates ± drugs; the worms were not transferred to new plates from adult day 10 to avoid physical injury of the sick worms during transferring. no FUDR was added. Animals were scored every 2 days. Worms that were nonresponsive to touch were considered dead (score 1). Missing worms or worms that died due to matricidal hatching, desiccation, or gonad extrusion were censored (score 0). The data were then represented by smoothed Kaplan–Meier survival curves, with statistical significance determined using the log‐rank test. *p* ≤ 0.05 was considered significant.

### 4.4 *Caenorhabditis elegans* healthspan analysis

Methods to quantify healthspan in *C*. *elegans* have been described previously (Hahm et al., 2015; Restif et al., 2014). Healthspan was assessed by maximum velocity, pharyngeal pumping, and swimming. To measure maximum velocity, we adapted a method previously described by Hahm et al. (2015). On the designated day, up to 10 worms were transferred to unseeded NGM plates. The movement was recorded over a span of 30 s using a stereomicroscope (Unitron 2850) and a charge‐coupled device camera (Infinity 2, Lumenera). Videos were analyzed using ImageJ software and the wMTrck Batch (http://www.phage.dk/plugins) plugin. To assess pharyngeal pumping, worms were assayed at the adult D6 stage. Contractions of the pharynx were manually counted over a period of 3 s in a double‐blinded manner. At least 30 worms were measured per group, and three independent experiments were conducted. Swimming analysis was conducted as described previously (Fang et al., 2017). At D8, individual worms were transferred from treatment plates onto a 6 cm dish containing 1 mL of M9 buffer. After an acclimatization period of ~1 s, the number of body bends was scored over a period of 30 s.

### 4.5 *Caenorhabditis elegans* mitochondrial networking

To visualize the mitochondrial networking in the *C*.* elegans* body wall muscle tissue, *csa*‐*1(tm4539)*, *csb*‐*1(ok2335)*, and *csa*‐*1(tm4539)*;*csb*‐*1(ok2335)* mutants were crossed to CB5600 *ccIs4251 [(pSAK2) myo*‐*3p*::*GFP*::*LacZ*::*NLS* + *(pSAK4)* *myo*‐*3p*::*mitochondrial GFP* + *dpy*‐*20(*+*)] I*, which is obtained from the Caenorhabditis Genetics Center (CGC). Worms were transferred to drug‐treated plates from the late L4 stages until day 7 unless otherwise noted. The mitochondrial network of randomly‐selected muscle cells between the pharynx and the vulva were imaged using a 40x objective lens. 2–4 muscle cells/worm were analyzed. Images were acquired with a ZEISS LSM 710 confocal microscope and analyzed as described previously (Fang et al., 2016, 2017). Imaging and scoring for mitochondrial networking integrity were performed by two different investigators in a double‐blinded manner. Mitochondrial networking was assessed using an arbitrary scale from 1–5. Healthy, organized mitochondrial networks in which the mitochondria are longer, less circular, aligned with the muscle fibers, and with reduced interconnecting mitochondrial strings received a score of 5 (Fang et al., 2017). The mitochondrial networking score was reduced if the mitochondria appeared disorganized and consisted of more globular mitochondria forming a network of irregular shapes. A total of 29–30 muscle cells from each group of worms (N = 10 worms/group) were collected.

### 4.6 *Caenorhabditis elegans* mitophagy

To visualize mitophagy in worms, the mitophagy reporter strain *N2*;*Ex(pmyo*‐*3∷dsred∷lgg*‐*1*;*pdct*‐*1∷dct*‐*1∷gfp)* was crossed with *csa*‐*1(tm4539)*, *csb*‐*1(ok2335)* and *csa*‐*1(tm4539)*; *csb*‐*1(ok2335) worms*. The muscle cells were imaged at D1 and D6 under a ZEISS LSM 710 confocal microscope as described previously (Palikaras et al., 2015). A total of 3‐6 worms/group were imaged. Zeiss LSM Image Examiner was used to measure the colocalization coefficient between DSRED∷DCT1 and LGG1∷GFP.

### 4.7 Electron microscopy

Electron microscopy (EM) was performed by Electron Microscopy Bioservices. Worm were washed with M9 buffer, cut with a scalpel blade on a petri dish, and then fixed in ice‐cold EM fixative buffer (2.5% glutaraldehyde, 2.0% paraformaldehyde in Millonig's Sodium Phosphate Buffer) for 2 h. Following rinsing with cold 1X PBS, worms were embedded in 2% agarose for ultrathin sectioning and microscopy. Ultrathin section analysis was performed on an electron microscope (Tecnai Spirit Twin Transmission; FEI) at 80 kV. The percent damaged mitochondria were counted from 19 random images. The mitochondrial length, area, and width were measured using ImageJ, n > 150.

### 4.8 Cockayne syndrome cerebellum samples

Human tissues were obtained from the NIH NeuorBioBank's Brain and Tissue repository at the University of Maryland, Baltimore. The patients and their ID numbers are listed in Tables S1 and S3. No correlation between postmortem interval (PMI) and RNA integrity was observed in CS brain and non‐CS controls (Figure S11). CS patients are clinically diagnosed.

### 4.9 Microarray analysis

RNA was purified from cerebellum samples from Cockayne patients and age‐ and gender‐matched healthy individuals by using the Nucleospin RNA isolation kit (catalog no. 740955.250; Macherey‐Nagel) with initial quantitation conducted using a NanoDrop ND‐1000 spectrophotometer (Thermo Fisher Scientific). A 2100 Bioanalyzer (Agilent Technologies) was used to confirm the quality of the RNA. The microarray was performed by the Gene Expression and Genomics Unit core facility (NIA) and analyzed using DIANE 6.0 software. The complete set was tested for Geneset enrichment using parametric analysis of gene set enrichment (PAGE). Detailed data analysis was performed as reported previously (Scheibye‐Knudsen et al., 2014). The samples (CS2 and CS4R) which clustered more closely with the controls (WT) than the CS patients were removed from further array analysis. The sample CS7x was identified as an outlier during quality analysis and excluded from the group analysis.

For microarray analysis of nematodes, N2, *csa*‐*1(tm4539)*, *csb*‐*1(ok2335)*, *csa*‐*1(tm4539)*; *csb*‐*1(ok2335)* worms were exposed to 500 µM NR from the L4 stage and whole‐body tissue was collected at adult day 7 for RNA extraction (Qiagen RNeasy mini kit with a QIAcube machine). Three biological replicates were prepared for each group. The Agilent kits were used for *C*.* elegans* microarray with the reagents including *C*.* elegans* (V2) Gene Expression Microarray (# G2519F‐020186), Low Input Quick Amp Labeling Kit (one color, #5190‐2305), Hybridization Backing Kit (# G2534‐60012), and Gene Expression Hybridization Kit (#5188‐5242). The microarray was performed and analyzed at the National Institute on Aging (NIA) Gene Expression and Genomics Unit using DIANE 6.0 software. Raw microarray data were log‐transformed to yield Z‐scores. The Z‐ratio was calculated as the difference between the observed gene Z‐scores for the experimental and the control comparisons divided by the standard deviation associated with the distribution of these differences. Z‐ratio ±1.5 with a *p*‐value ≤0.05, 0.05 false discovery rate (FDR), and average signal intensity of comparison greater than zero were used as cutoff values (the false discovery rate is calculated by the Benjamini–Hochberg procedure). The gene expression Z‐ratio values were then used as input to perform Parametric Analysis of Gene Set Enrichment (PAGE) testing (Kim & Volsky, 2005). The accession number for both array data is GSE144558.

### 4.10 Cell culture and cell lines

The normal human fetal lung primary fibroblasts were received from Coriell Institute (ID #I90‐83) and were cultured in Dulbecco's modified Eagle medium (DMEM) supplemented with 10% (vol/vol) FBS and 1% penicillin/streptomycin in a humidified chamber under 5% (vol/vol) CO2 at 37°C.

### 4.11 siRNA knockdown

siERCC6 (5′‐CCACUACAAUAGCUUCAAGACAGCC‐3′), siERCC8 (5′‐GGAGAACAGAUAACUAUGCUUAAGG −3′) and siRNA duplex control (Origene) was diluted with DMEM to a final concentration of 20 nM, mixed with INTERFERin (Polyplus transfection), incubated for 15 min at room temperature, and transfected into primary cells target according to the manufacturer's instructions. Twenty‐four hours after transfection, the media was replaced with FBS media. Forty‐eight hours after transfection, the cells were treated with NMN (500 µM) or water as a vehicle for 24 h and lysed for western blotting.

### 4.12 Immunoblotting

Cells were lysed in RIPA buffer (#9806S; Cell Signaling) with Halt™ Protease and Phosphatase Inhibitor Cocktail (100X) (#78444, Thermo Fisher Scientific) unless indicated otherwise. Antibodies were used according to the manufacturer's instructions to detect the following antigens: AMPK (Cell signaling, #5831), p‐AMPK (Thr172) (Cell signaling, #2535), pULK1 (Ser555, Cell signaling, #5869), ULK1 (Cell signaling, #6439), p62 (Cell signaling, #39749), β‐actin (Santa Cruz, #sc‐1616), DRP1 (Cell signaling, #8570), p‐DRP‐1 (S616, Cell signaling, #3455), Beclin1 (Cell signaling, #3738), mitofusin 2 (Cell signaling, #9482), total OXHOS rodent cocktail (Abcam, ab110413), SOD‐1 (Cell signaling, #2770), SOD‐2 (Enzo Life Sciences, #ADI‐SOD‐110‐F), γ‐H2AX (Santa Cruz, #sc‐517348), and GAPDH (Abclonal, #AC027).

### 4.13 Animals

Mouse models of CSB^m/m^ (mice carrying a premature stop codon in exon 5 to mimic the K337 stop truncation mutation from CS patient CS1AN) (Horst et al., 1997) and wild type (WT) on a C57BL/6 J background in the age ranges of 20–26 weeks (young), 52–62 weeks (mid‐age), and 98–11 weeks (old) of age) were used for the grip strength studies. All animal protocols were approved by the appropriate institutional animal care and use committee of the National Institute on Aging.

### 4.14 Grip strength

The forelimbs of each mouse were tested for grip strength by pulling on a wire attached to a Chatillon DFE002 force gauge (Chatillon Force Measurement Systems). Five pulls were performed for each mouse, and the mean of the recordings was determined as a final score.

### 4.15 Statistical analysis

All statistical analyses were performed with GraphPad Prism (GraphPad Software, Inc.). For multiple samples with two groups, two‐way ANOVA with Tukey's post hoc test was used to determine significant differences. One‐way ANOVA with Tukey's post hoc test was used to determine significant differences across multiple samples. t‐test was used to compare two groups. The log‐rank test was used to determine statistical significance for survival assays, and *p* ≤ 0.05 was considered significant.

## 5 ETHICS APPROVAL AND CONSENT TO PARTICIPATE

All animal protocols were approved by the appropriate institutional animal care and use committee of the National Institute on Aging. Human tissues were obtained from the NIH NeuorBioBank's Brain and Tissue repository at the University of Maryland, Baltimore.

## CONFLICT OF INTEREST

V.A.B. and E.F.F. have CRADA agreements with and receive Nicotinamide Riboside from ChromaDex. E.F.F. is a consultant to Aladdin Healthcare Technologies and the Vancouver Dementia Prevention Centre.

## AUTHOR CONTRIBUTIONS

E.F.F. and M.N.O. planned the experiments. E.M.F., M.N.O., E.F.F., and V.T. performed the experiments; V.A.B. and D.L.C supervised the findings of the work; D.L.C, M.N.O., and V.T. wrote the manuscript with support from E.M.F. All authors discussed the results and contributed to the final manuscript.

## Supporting information

Appendix S1

Data 1

## Data Availability

The datasets used and/or analyzed during the current study are available from the corresponding author on reasonable request.

## References

[acel13268-bib-0001] Aamann, M. D. , Sorensen, M. M. , Hvitby, C. , Berquist, B. R. , Muftuoglu, M. , Tian, J. , Souza‐Pinto, N. C. , Scheibye‐Knudsen, M. , Wilson, D. M. , Stevnsner, T. , & Bohr, V. A. (2010). Cockayne syndrome group B protein promotes mitochondrial DNA stability by supporting the DNA repair association with the mitochondrial membrane. FASEB Journal, 24, 2334–2346. 10.1096/fj.09-147991 20181933 PMC2887265

[acel13268-bib-0002] Andrade, L. N. , Nathanson, J. L. , Yeo, G. W. , Menck, C. F. , & Muotri, A. R. (2012). Evidence for premature aging due to oxidative stress in iPSCs from Cockayne syndrome. Human Molecular Genetics, 21, 3825–3834. 10.1093/hmg/dds211 22661500 PMC3412382

[acel13268-bib-0003] Babu, V. , Hofmann, K. , & Schumacher, B. A. C. (2014). elegans homolog of the Cockayne syndrome complementation group A gene. DNA Repair, 24, 57–62. 10.1016/j.dnarep.2014.09.011 25453470 PMC4255241

[acel13268-bib-0004] Berquist, B. R. , Canugovi, C. , Sykora, P. , Wilson, D. M. , & Bohr, V. A. (2012). Human Cockayne syndrome B protein reciprocally communicates with mitochondrial proteins and promotes transcriptional elongation. Nucleic Acids Research, 40, 8392–8405. 10.1093/nar/gks565 22743267 PMC3458532

[acel13268-bib-0005] Bianco, J. N. , & Schumacher, B. (2018). MPK‐1/ERK pathway regulates DNA damage response during development through DAF‐16/FOXO. Nucleic Acids Research, 46, 6129–6139. 10.1093/nar/gky404 29788264 PMC6159517

[acel13268-bib-0006] Brenner, S. (1974). The genetics of *Caenorhabditis elegans* . Genetics, 77, 71–94. 4366476 10.1093/genetics/77.1.71 PMC1213120

[acel13268-bib-0007] Canto, C. , Gerhart‐Hines, Z. , Feige, J. N. , Lagouge, M. , Noriega, L. , Milne, J. C. , Elliott, P. J. , Puigserver, P. , & Auwerx, J. (2009). AMPK regulates energy expenditure by modulating NAD+ metabolism and SIRT1 activity. Nature, 458, 1056–1060. 10.1038/nature07813 19262508 PMC3616311

[acel13268-bib-0008] Egan, D. F. , Shackelford, D. B. , Mihaylova, M. M. , Gelino, S. , Kohnz, R. A. , Mair, W. , Vasquez, D. S. , Joshi, A. , Gwinn, D. M. , Taylor, R. , Asara, J. M. , Fitzpatrick, J. , Dillin, A. , Viollet, B. , Kundu, M. , Hansen, M. , & Shaw, R. J. (2011). Phosphorylation of ULK1 (hATG1) by AMP‐activated protein kinase connects energy sensing to mitophagy. Science, 331, 456–461. 10.1126/science.1196371 21205641 PMC3030664

[acel13268-bib-0009] Epanchintsev, A. , Costanzo, F. , Rauschendorf, M.‐A. , Caputo, M. , Ye, T. , Donnio, L.‐M. , Proietti‐de‐Santis, L. , Coin, F. , Laugel, V. , & Egly, J.‐M. (2017). Cockayne's Syndrome A and B proteins regulate transcription arrest after genotoxic stress by promoting ATF3 degradation. Molecular Cell, 68, 1054–1066. 10.1016/j.molcel.2017.11.009 29225035

[acel13268-bib-0010] Fang, E. F. , Hou, Y. , Lautrup, S. , Jensen, M. B. , Yang, B. , SenGupta, T. , Caponio, D. , Khezri, R. , Demarest, T. G. , Aman, Y. , Figueroa, D. , Morevati, M. , Lee, H.‐J. , Kato, H. , Kassahun, H. , Lee, J.‐H. , Filippelli, D. , Okur, M. N. , Mangerich, A. , … Bohr, V. A. (2019). NAD(+) augmentation restores mitophagy and limits accelerated aging in Werner syndrome. Nature Communications, 10, 5284 10.1038/s41467-019-13172-8 PMC6872719 31754102

[acel13268-bib-0011] Fang, E. F. , Hou, Y. , Palikaras, K. , Adriaanse, B. A. , Kerr, J. S. , Yang, B. , Lautrup, S. , Hasan‐Olive, M. M. , Caponio, D. , Dan, X. , Rocktäschel, P. , Croteau, D. L. , Akbari, M. , Greig, N. H. , Fladby, T. , Nilsen, H. , Cader, M. Z. , Mattson, M. P. , Tavernarakis, N. , & Bohr, V. A. (2019). Mitophagy inhibits amyloid‐beta and tau pathology and reverses cognitive deficits in models of Alzheimer's disease. Nature Neuroscience, 22, 401–412. 10.1038/s41593-018-0332-9 30742114 PMC6693625

[acel13268-bib-0012] Fang, E. F. , Kassahun, H. , Croteau, D. L. , Scheibye‐Knudsen, M. , Marosi, K. , Lu, H. , Shamanna, R. A. , Kalyanasundaram, S. , Bollineni, R. C. , Wilson, M. A. , Iser, W. B. , Wollman, B. N. , Morevati, M. , Li, J. , Kerr, J. S. , Lu, Q. , Waltz, T. B. , Tian, J. , Sinclair, D. A. … Bohr, V. A. (2016). NAD(+) Replenishment improves lifespan and healthspan in Ataxia Telangiectasia Models via mitophagy and DNA Repair. Cell Metabolism, 24, 566–581. 10.1016/j.cmet.2016.09.004 27732836 PMC5777858

[acel13268-bib-0013] Fang, E. F. , Scheibye‐Knudsen, M. , Brace, L. E. , Kassahun, H. , SenGupta, T. , Nilsen, H. , Mitchell, J. R. , Croteau, D. L. , & Bohr, V. A. (2014). Defective mitophagy in XPA via PARP‐1 hyperactivation and NAD(+)/SIRT1 reduction. Cell, 157, 882–896. 10.1016/j.cell.2014.03.026 24813611 PMC4625837

[acel13268-bib-0014] Fang, E. F. , Waltz, T. B. , Kassahun, H. , Lu, Q. , Kerr, J. S. , Morevati, M. , Fivenson, E. M. , Wollman, B. N. , Marosi, K. , Wilson, M. A. , Iser, W. B. , Eckley, D. M. , Zhang, Y. , Lehrmann, E. , Goldberg, I. G. , Scheibye‐Knudsen, M. , Mattson, M. P. , Nilsen, H. , Bohr, V. A. , & Becker, K. G. (2017). Tomatidine enhances lifespan and healthspan in C. elegans through mitophagy induction via the SKN‐1/Nrf2 pathway. Scientific Reports, 7(1), 46208 10.1038/srep46208 28397803 PMC5387417

[acel13268-bib-0015] Gilmour, B. C. , Gudmundsrud, R. , Frank, J. , Hov, A. , Lautrup, S. , Aman, Y. , Røsjø, H. , Brenner, C. , Ziegler, M. , Tysnes, O. B. , Tzoulis, C. , Omland, T. , Søraas, A. , Holmøy, T. , Bergersen, L. H. , Storm‐Mathisen, J. , Nilsen, H. , & Fang, E. F. (2020). Targeting NAD(+) in translational research to relieve diseases and conditions of metabolic stress and ageing. Mechanisms of Ageing and Development, 111208, 10.1016/j.mad.2020.111208 31953124

[acel13268-bib-0016] Gorgels, T. G. , van der Pluijm, I. , Brandt, R. M. , Garinis, G. A. , van Steeg, H. , van den Aardweg, G. , Jansen, G. H. , Ruijter, J. M. , Bergen, A. A. , van Norren, D. , Hoeijmakers, J. H. , & van der Horst, G. T. (2007). Retinal degeneration and ionizing radiation hypersensitivity in a mouse model for Cockayne syndrome. Molecular and Cellular Biology, 27, 1433–1441. 10.1128/MCB.01037-06 17145777 PMC1800713

[acel13268-bib-0017] Hahm, J. H. , Kim, S. , DiLoreto, R. , Shi, C. , Lee, S. J. , Murphy, C. T. , & Nam, H. G. (2015). C. elegans maximum velocity correlates with healthspan and is maintained in worms with an insulin receptor mutation. Nature Communications, 6(1), 8919. 10.1038/ncomms9919 PMC4656132 26586186

[acel13268-bib-0018] Johri, A. , & Beal, M. F. (2012). Mitochondrial dysfunction in neurodegenerative diseases. Journal of Pharmacology and Experimental Therapeutics, 342, 619–630. 10.1124/jpet.112.192138 PMC3422529 22700435

[acel13268-bib-0019] Karikkineth, A. C. , Scheibye‐Knudsen, M. , Fivenson, E. , Croteau, D. L. , & Bohr, V. A. (2017). Cockayne syndrome: Clinical features, model systems and pathways. Ageing Research Reviews, 33, 3–17. 10.1016/j.arr.2016.08.002 27507608 PMC5195851

[acel13268-bib-0020] Kim, S. Y. , & Volsky, D. J. (2005). PAGE: parametric analysis of gene set enrichment. BMC Bioinformatics, 6, 144 10.1186/1471-2105-6-144 15941488 PMC1183189

[acel13268-bib-0021] Lake, R. J. , Boetefuer, E. L. , Tsai, P.‐F. , Jeong, J. , Choi, I. , Won, K.‐J. , & Fan, H.‐Y. (2014). The sequence‐specific transcription factor c‐Jun Targets Cockayne Syndrome protein B to regulate transcription and chromatin structure. PLoS Genetics, 10, e1004284 10.1371/journal.pgen.1004284 24743307 PMC3990521

[acel13268-bib-0022] Lans, H. , Lindvall, J. M. , Thijssen, K. , Karambelas, A. E. , Cupac, D. , Fensgård, Ø. , Jansen, G. , Hoeijmakers, J. H. J. , Nilsen, H. , & Vermeulen, W. (2013). DNA damage leads to progressive replicative decline but extends the life span of long‐lived mutant animals. Cell Death and Differentiation, 20, 1709–1718. 10.1038/cdd.2013.126 24013725 PMC3824592

[acel13268-bib-0023] Lans, H. , & Vermeulen, W. (2011). Nucleotide excision repair in *Caenorhabditis elegans* . Molecular Biology International, 2011, 542795 10.4061/2011/542795 22091407 PMC3195855

[acel13268-bib-0024] Li, Y. , & Chen, Y. (2019). AMPK and autophagy. Advances in Experimental Medicine and Biology, 1206, 85–108. 10.1007/978-981-15-0602-4_4 31776981

[acel13268-bib-0025] Mayne, L. V. , & Lehmann, A. R. (1982). Failure of RNA synthesis to recover after UV irradiation: An early defect in cells from individuals with Cockayne's syndrome and xeroderma pigmentosum. Cancer Research, 42, 1473–1478. 6174225

[acel13268-bib-0026] Mitchell, D. H. , Stiles, J. W. , Santelli, J. , & Sanadi, D. R. (1979). Synchronous growth and aging of Caenorhabditis elegans in the presence of fluorodeoxyuridine. Journal of Gerontology, 34, 28–36. 153363 10.1093/geronj/34.1.28

[acel13268-bib-0027] Okur, M. N. , Lee, J.‐H. , Osmani, W. , Kimura, R. , Demarest, T. G. , Croteau, D. L. , & Bohr, V. A. (2020). Cockayne syndrome group A and B proteins function in rRNA transcription through nucleolin regulation. Nucleic Acids Research, 48(5), 2473–2485. 10.1093/nar/gkz1242 31970402 PMC7049711

[acel13268-bib-0028] Okur, M. N. , Mao, B. , Kimura, R. , Haraczy, S. , Fitzgerald, T. , Edwards‐Hollingsworth, K. , Tian, J. , Osmani, W. , Croteau, D. L. , Kelley, M. W. , & Bohr, V. A. (2020). Short‐term NAD(+) supplementation prevents hearing loss in mouse models of Cockayne syndrome. NPJ Aging and Mechanisms of Disease, 6, 1 10.1038/s41514-019-0040-z 31934345 PMC6946667

[acel13268-bib-0029] Palikaras, K. , Lionaki, E. , & Tavernarakis, N. (2015). Coordination of mitophagy and mitochondrial biogenesis during ageing in *C‐elegans* . Nature, 521, 525–U241. 10.1038/nature14300 25896323

[acel13268-bib-0030] Pascucci, B. , Fragale, A. , Marabitti, V. , Leuzzi, G. , Calcagnile, A. S. , Parlanti, E. , Franchitto, A. , Dogliotti, E. , & D'Errico, M. (2018). CSA and CSB play a role in the response to DNA breaks. Oncotarget, 9, 11581–11591. 10.18632/oncotarget.24342 29545921 PMC5837770

[acel13268-bib-0031] Raimundo, N. , Fernandez‐Mosquera, L. , Yambire, K. F. , & Diogo, C. V. (2016). Mechanisms of communication between mitochondria and lysosomes. The International Journal of Biochemistry & Cell Biology, 79, 345–349. 10.1016/j.biocel.2016.08.020 27613573

[acel13268-bib-0032] Restif, C. , Ibáñez‐Ventoso, C. , Vora, M. M. , Guo, S. , Metaxas, D. , & Driscoll, M. (2014). CeleST: computer vision software for quantitative analysis of C. elegans swim behavior reveals novel features of locomotion. PLoS Computational Biology, 10, e1003702. 25033081 10.1371/journal.pcbi.1003702 PMC4102393

[acel13268-bib-0033] Scheibye‐Knudsen, M. , Mitchell, S. J. , Fang, E. F. , Iyama, T. , Ward, T. , Wang, J. , Dunn, C. A. , Singh, N. , Veith, S. , Hasan‐Olive, M. M. , Mangerich, A. , Wilson, M. A. , Mattson, M. P. , Bergersen, L. H. , Cogger, V. C. , Warren, A. , Le Couteur, D. G. , Moaddel, R. , Wilson, D. M. 3rd , … Bohr, V. A. (2014). A high‐fat diet and NAD(+) activate Sirt1 to rescue premature aging in cockayne syndrome. Cell Metabolism, 20, 840–855. 10.1016/j.cmet.2014.10.005 25440059 PMC4261735

[acel13268-bib-0034] Scheibye‐Knudsen, M. , Ramamoorthy, M. , Sykora, P. , Maynard, S. , Lin, P. C. , Minor, R. K. , Wilson, D. M. 3rd , Cooper, M. , Spencer, R. , de Cabo, R. , Croteau, D. L. , & Bohr, V. A. (2012). Cockayne syndrome group B protein prevents the accumulation of damaged mitochondria by promoting mitochondrial autophagy. Journal of Experimental Medicine, 209, 855–869. 10.1084/jem.20111721 PMC3328359 22473955

[acel13268-bib-0035] Scheibye‐Knudsen, M. , Tseng, A. , Borch Jensen, M. , Scheibye‐Alsing, K. , Fang, E. F. , Iyama, T. , Bharti, S. K. , Marosi, K. , Froetscher, L. , Kassahun, H. , Eckley, D. M. , Maul, R. W. , Bastian, P. , De, S. , Ghosh, S. , Nilsen, H. , Goldberg, I. G. , Mattson, M. P. , Wilson, D. M. 3rd , … Bohr, V. A. (2016). Cockayne syndrome group A and B proteins converge on transcription‐linked resolution of non‐B DNA. Proceedings of the National Academy of Sciences, 113, 12502–12507. 10.1073/pnas.1610198113 PMC5098674 27791127

[acel13268-bib-0036] Stevnsner, T. , Nyaga, S. , de Souza‐Pinto, N. C. , van der Horst, G. T. , Gorgels, T. G. , Hogue, B. A. , Thorslund, T. , & Bohr, V. A. (2002). Mitochondrial repair of 8‐oxoguanine is deficient in Cockayne syndrome group B. Oncogene, 21, 8675–8682. 10.1038/sj.onc.1205994 12483520

[acel13268-bib-0037] Toyama, E. Q. , Herzig, S. , Courchet, J. , Lewis, T. L. , Loson, O. C. , Hellberg, K. , Young, N. P. , Chen, H. , Polleux, F. , Chan, D. C. , & Shaw, R. J. (2016). Metabolism. AMP‐activated protein kinase mediates mitochondrial fission in response to energy stress. Science 351, 275–281. 10.1126/science.aab4138 26816379 PMC4852862

[acel13268-bib-0038] van der Horst, G. T. ., Meira, L. , Gorgels, T. G. , de Wit, J. , Velasco‐Miguel, S. , Richardson, J. A. , Kamp, Y. , Vreeswijk, M. P. , Smit, B. , Bootsma, D. , Hoeijmakers, J. H. , & Friedberg, E. C. (2002). UVB radiation‐induced cancer predisposition in Cockayne syndrome group A (Csa) mutant mice. DNA Repair (Amst), 1, 143–157. 12509261 10.1016/s1568-7864(01)00010-6

[acel13268-bib-0039] van der Horst, G. T. , van Steeg, H. , Berg, R. J. W. , van Gool, A. J. , de Wit, J. , Weeda, G. , Morreau, H. , Beems, R. B. , van Kreijl, C. F. , de Gruijl, F. R. , Bootsma, D. & Hoeijmakers, J. H. J. (1997). Defective transcription‐coupled repair in Cockayne syndrome B mice is associated with skin cancer predisposition. Cell, 89, 425–435. 9150142 10.1016/s0092-8674(00)80223-8

[acel13268-bib-0040] Wang, C. , & Youle, R. (2016). Cell biology: Form follows function for mitochondria. Nature, 530, 288–289. 10.1038/530288a 26887490

[acel13268-bib-0041] Wang, Y. , Chakravarty, P. , Ranes, M. , Kelly, G. , Brooks, P. J. , Neilan, E. , Stewart, A. , Schiavo, G. , & Svejstrup, J. Q. (2014). Dysregulation of gene expression as a cause of Cockayne syndrome neurological disease. Proceedings of the National Academy of Sciences, 111, 14454–14459. 10.1073/pnas.1412569111 PMC4210037 25249633

[acel13268-bib-0042] Yu, S. B. , & Pekkurnaz, G. (2018). Mechanisms orchestrating mitochondrial dynamics for energy homeostasis. Journal of Molecular Biology, 430, 3922–3941. 10.1016/j.jmb.2018.07.027 30089235 PMC6186503

[acel13268-bib-0043] Zhang, J. , Wang, C. , Shi, H. , Wu, D. , & Ying, W. (2018). Extracellular degradation into adenosine and the activities of adenosine kinase and AMPK Mediate Extracellular NAD(+)‐produced increases in the adenylate pool of BV2 microglia under basal conditions. Frontiers in Cellular Neuroscience, 12, 343 10.3389/fncel.2018.00343 30405351 PMC6200843

