# Supplementary material for: Cockayne syndrome proteins CSA and CSB maintain mitochondrial homeostasis through NAD+ signaling
Source: Aging Cell. 2020 Nov 9;19(12):e13268. doi: 10.1111/acel.13268 (PMC7744955; doi:10.1111/acel.13268)
Supplement: Supplementary file 1 — Appendix S1 [file ACEL-19-e13268-s001.pdf]

Cockayne syndrome proteins CSA and CSB maintain mitochondrial homeostasis through NAD^+ signaling

Figure 1

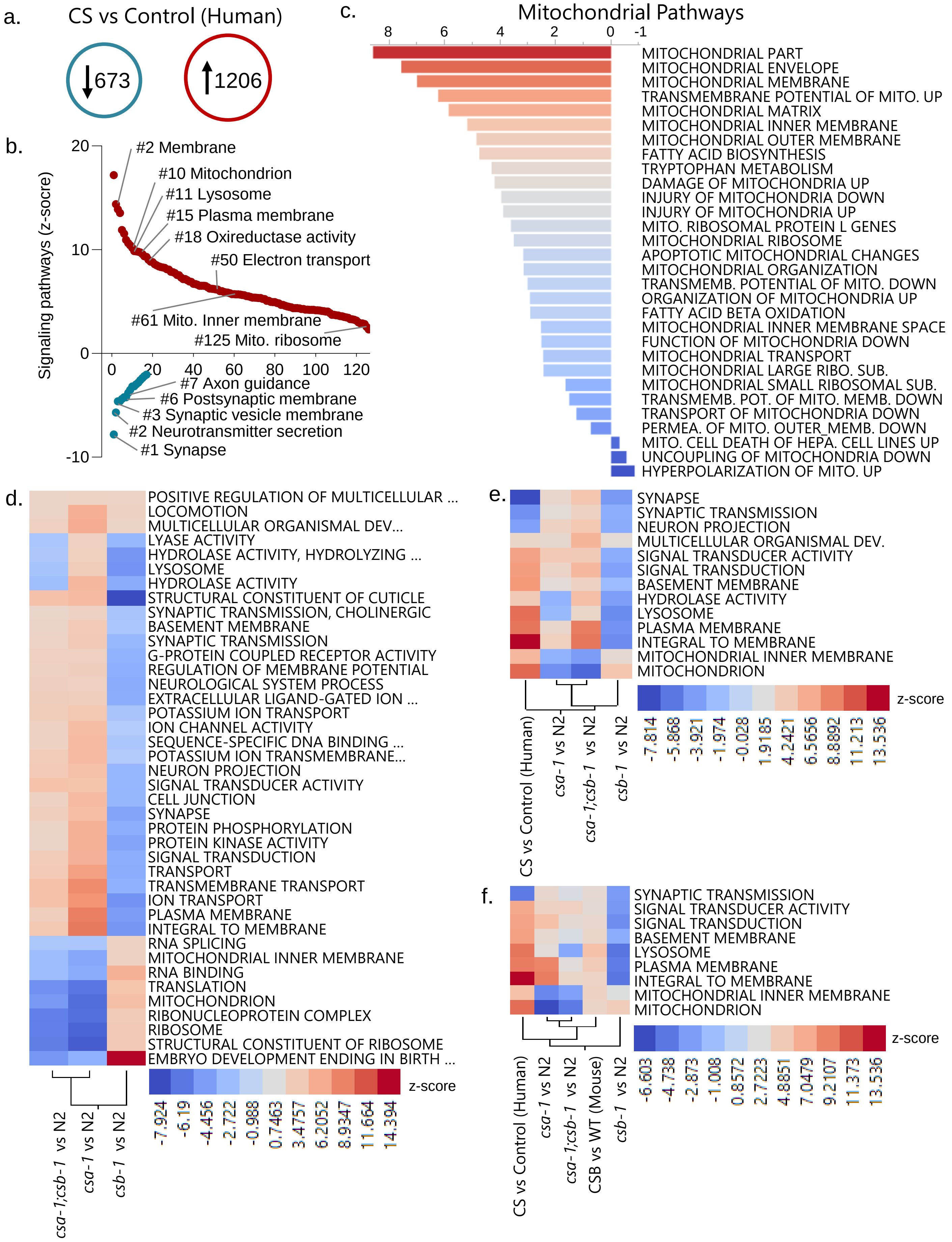

a.
CS vs Control (Human)
673
1206
b.
Signaling pathways (z-socre)
#2 Membrane
#10 Mitochondrion
#11 Lysosome
#15 Plasma membrane
#18 Oxireductase activity
#50 Electron transport
#61 Mito. Inner membrane
#125 Mito. ribosome
#7 Axon guidance
#6 Postsynaptic membrane
#3 Synaptic vesicle membrane
#2 Neurotransmitter secretion
#1 Synapse
c.
Mitochondrial Pathways
MITOCHONDRIAL PART
MITOCHONDRIAL ENVELOPE
MITOCHONDRIAL MEMBRANE
TRANSMEMBRANE POTENTIAL OF MITO. UP
MITOCHONDRIAL MATRIX
MITOCHONDRIAL INNER MEMBRANE
MITOCHONDRIAL OUTER MEMBRANE
FATTY ACID BIOSYNTHESIS
TRYPTOPHAN METABOLISM
DAMAGE OF MITOCHONDRIA UP
INJURY OF MITOCHONDRIA DOWN
INJURY OF MITOCHONDRIA UP
MITO. RIBOSOMAL PROTEIN L GENES
MITOCHONDRIAL RIBOSOME
APOPTOTIC MITOCHONDRIAL CHANGES
MITOCHONDRIAL ORGANIZATION
TRANSMEMB. POTENTIAL OF MITO. DOWN
ORGANIZATION OF MITOCHONDRIA UP
FATTY ACID BETA OXIDATION
MITOCHONDRIAL INNER MEMBRANE SPACE
FUNCTION OF MITOCHONDRIA DOWN
MITOCHONDRIAL TRANSPORT
MITOCHONDRIAL LARGE RIBO. SUB.
MITOCHONDRIAL SMALL RIBOSOMAL SUB.
TRANSMEMB. POT. OF MITO. MEMB. DOWN
TRANSPORT OF MITOCHONDRIA DOWN
PERMEA. OF MITO. OUTER_MEMB. DOWN
MITO. CELL DEATH OF HEPA. CELL LINES UP
UNCOUPLING OF MITOCHONDRIA DOWN
HYPERPOLARIZATION OF MITO. UP
d.
POSITIVE REGULATION OF MULTICELLULAR ...
LOCOMOTION
MULTICELLULAR ORGANISMAL DEV...
LYASE ACTIVITY
HYDROLASE ACTIVITY, HYDROLYZING ...
LYSOSOME
HYDROLASE ACTIVITY
STRUCTURAL CONSTITUENT OF CUTICLE
SYNAPTIC TRANSMISSION, CHOLINERGIC
BASEMENT MEMBRANE
SYNAPTIC TRANSMISSION
G-PROTEIN COUPLED RECEPTOR ACTIVITY
REGULATION OF MEMBRANE POTENTIAL
NEUROLOGICAL SYSTEM PROCESS
EXTRACELLULAR LIGAND-GATED ION ...
POTASSIUM ION TRANSPORT
ION CHANNEL ACTIVITY
SEQUENCE-SPECIFIC DNA BINDING ...
POTASSIUM ION TRANSMEMBRANE...
NEURON PROJECTION
SIGNAL TRANSDUCER ACTIVITY
CELL JUNCTION
SYNAPSE
PROTEIN PHOSPHORYLATION
PROTEIN KINASE ACTIVITY
SIGNAL TRANSDUCTION
TRANSPORT
TRANSMEMBRANE TRANSPORT
ION TRANSPORT
PLASMA MEMBRANE
INTEGRAL TO MEMBRANE
RNA SPLICING
MITOCHONDRIAL INNER MEMBRANE
RNA BINDING
TRANSLATION
MITOCHONDRION
RIBONUCLEOPROTEIN COMPLEX
RIBOSOME
STRUCTURAL CONSTITUENT OF RIBOSOME
EMBRYO DEVELOPMENT ENDING IN BIRTH ...
z-score
-7.924 -6.19 -4.456 -2.722 -0.988 0.7463 3.4757 6.2052 8.9347 11.664 14.394
csa-1;csb-1 vs N2
csa-1 vs N2
csb-1 vs N2
e.
SYNAPSE
SYNAPTIC TRANSMISSION
NEURON PROJECTION
MULTICELLULAR ORGANISMAL DEV.
SIGNAL TRANSDUCER ACTIVITY
SIGNAL TRANSDUCTION
BASEMENT MEMBRANE
HYDROLASE ACTIVITY
LYSOSOME
PLASMA MEMBRANE
INTEGRAL TO MEMBRANE
MITOCHONDRIAL INNER MEMBRANE
MITOCHONDRION
z-score
-7.814 -5.868 -3.921 -1.974 -0.028 1.9185 4.2421 6.5656 8.8892 11.213 13.536
CS vs Control (Human)
csa-1 vs N2
csa-1;csb-1 vs N2
csb-1 vs N2
f.
SYNAPTIC TRANSMISSION
SIGNAL TRANSDUCER ACTIVITY
SIGNAL TRANSDUCTION
BASEMENT MEMBRANE
LYSOSOME
PLASMA MEMBRANE
INTEGRAL TO MEMBRANE
MITOCHONDRIAL INNER MEMBRANE
MITOCHONDRION
z-score
-6.603 -4.738 -2.873 -1.008 0.8572 2.7223 4.8851 7.0479 9.2107 11.373 13.536
CS vs Control (Human)
csa-1 vs N2
csa-1;csb-1 vs N2
CSB vs WT (Mouse)
csb-1 vs N2

Figure 2

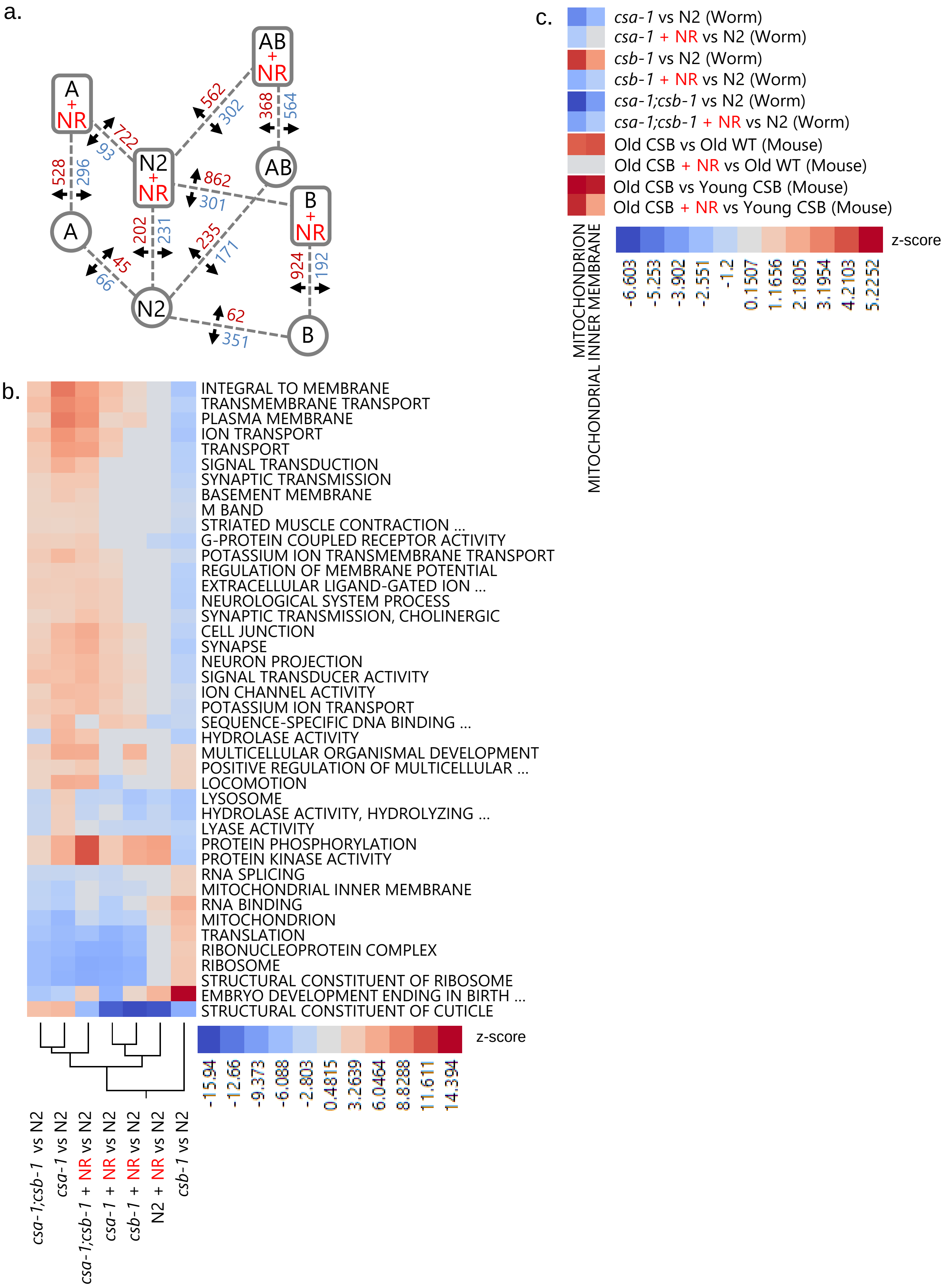
a.
A + NR
AB + NR
N2 + NR
AB
B + NR
A
N2
B
528
296
722
93
562
302
368
564
862
301
202
231
235
171
45
66
924
192
62
351
b.
INTEGRAL TO MEMBRANE
TRANSMEMBRANE TRANSPORT
PLASMA MEMBRANE
ION TRANSPORT
TRANSPORT
SIGNAL TRANSDUCTION
SYNAPTIC TRANSMISSION
BASEMENT MEMBRANE
M BAND
STRIATED MUSCLE CONTRACTION ...
G-PROTEIN COUPLED RECEPTOR ACTIVITY
POTASSIUM ION TRANSMEMBRANE TRANSPORT
REGULATION OF MEMBRANE POTENTIAL
EXTRACELLULAR LIGAND-GATED ION ...
NEUROLOGICAL SYSTEM PROCESS
SYNAPTIC TRANSMISSION, CHOLINERGIC
CELL JUNCTION
SYNAPSE
NEURON PROJECTION
SIGNAL TRANSDUCER ACTIVITY
ION CHANNEL ACTIVITY
POTASSIUM ION TRANSPORT
SEQUENCE-SPECIFIC DNA BINDING ...
HYDROLASE ACTIVITY
MULTICELLULAR ORGANISMAL DEVELOPMENT
POSITIVE REGULATION OF MULTICELLULAR ...
LOCOMOTION
LYSOSOME
HYDROLASE ACTIVITY, HYDROLYZING ...
LYASE ACTIVITY
PROTEIN PHOSPHORYLATION
PROTEIN KINASE ACTIVITY
RNA SPLICING
MITOCHONDRIAL INNER MEMBRANE
RNA BINDING
MITOCHONDRION
TRANSLATION
RIBONUCLEOPROTEIN COMPLEX
RIBOSOME
STRUCTURAL CONSTITUENT OF RIBOSOME
EMBRYO DEVELOPMENT ENDING IN BIRTH ...
STRUCTURAL CONSTITUENT OF CUTICLE
z-score
-15.94
-12.66
-9.373
-6.088
-2.803
0.4815
3.2639
6.0464
8.8288
11.611
14.394
csa-1;csb-1 vs N2
csa-1 vs N2
csa-1;csb-1 + NR vs N2
csa-1 + NR vs N2
csb-1 + NR vs N2
N2 + NR vs N2
csb-1 vs N2
c.
csa-1 vs N2 (Worm)
csa-1 + NR vs N2 (Worm)
csb-1 vs N2 (Worm)
csb-1 + NR vs N2 (Worm)
csa-1;csb-1 vs N2 (Worm)
csa-1;csb-1 + NR vs N2 (Worm)
Old CSB vs Old WT (Mouse)
Old CSB + NR vs Old WT (Mouse)
Old CSB vs Young CSB (Mouse)
Old CSB + NR vs Young CSB (Mouse)
MITOCHONDRION
MITOCHONDRIAL INNER MEMBRANE
z-score
-6.603
-5.253
-3.902
-2.551
-1.2
0.1507
1.1656
2.1805
3.1954
4.2103
5.2252

Figure 3

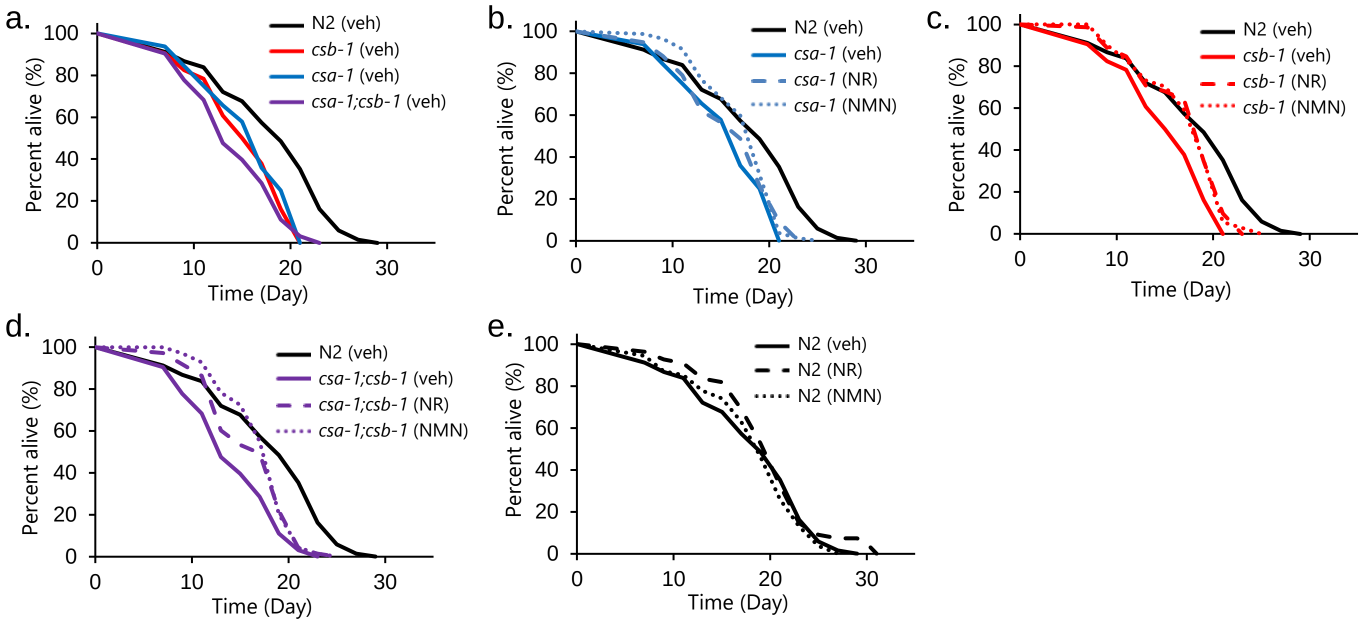

h.

Genotype and Treatment	Mean ±SEM	p-value	
		vs. N2	vs. Veh
N2 (veh)	18.3 ± 0.7		
N2 (NR)	19.7 ± 0.7	0.32	
N2 (NMN)	18.4 ± 0.7	0.58	
csa-1 (veh)	15.3 ± 0.5	<0.001	
csa-1 (NR)	16.3 ± 0.5		0.055
csa-1 (NMN)	17.6 ± 0.4		0.022
csb-1 (veh)	15.8 ± 0.6	<0.001	
csb-1 (NR)	17.4 ± 0.5		0.01
csb-1 (NMN)	17.4 ± 0.5		0.025
csa-1;csb-1 (veh)	14.3 ± 0.6	<0.001	
csa-1;csb-1 (NR)	16.3 ± 0.5		0.012
csa-1;csb-1 (NMN)	17.4 ± 0.4		<0.001

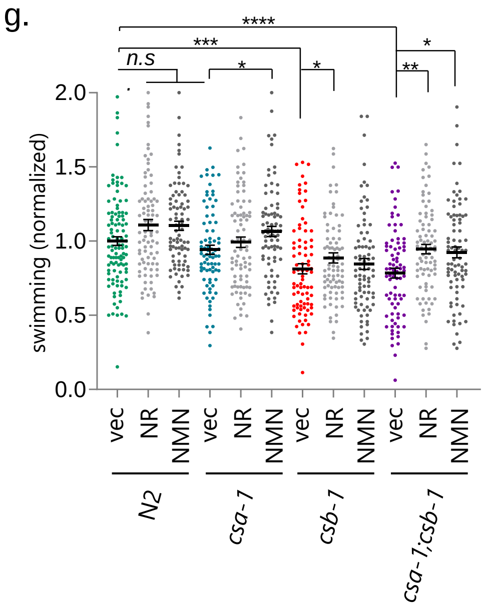

Figure 4

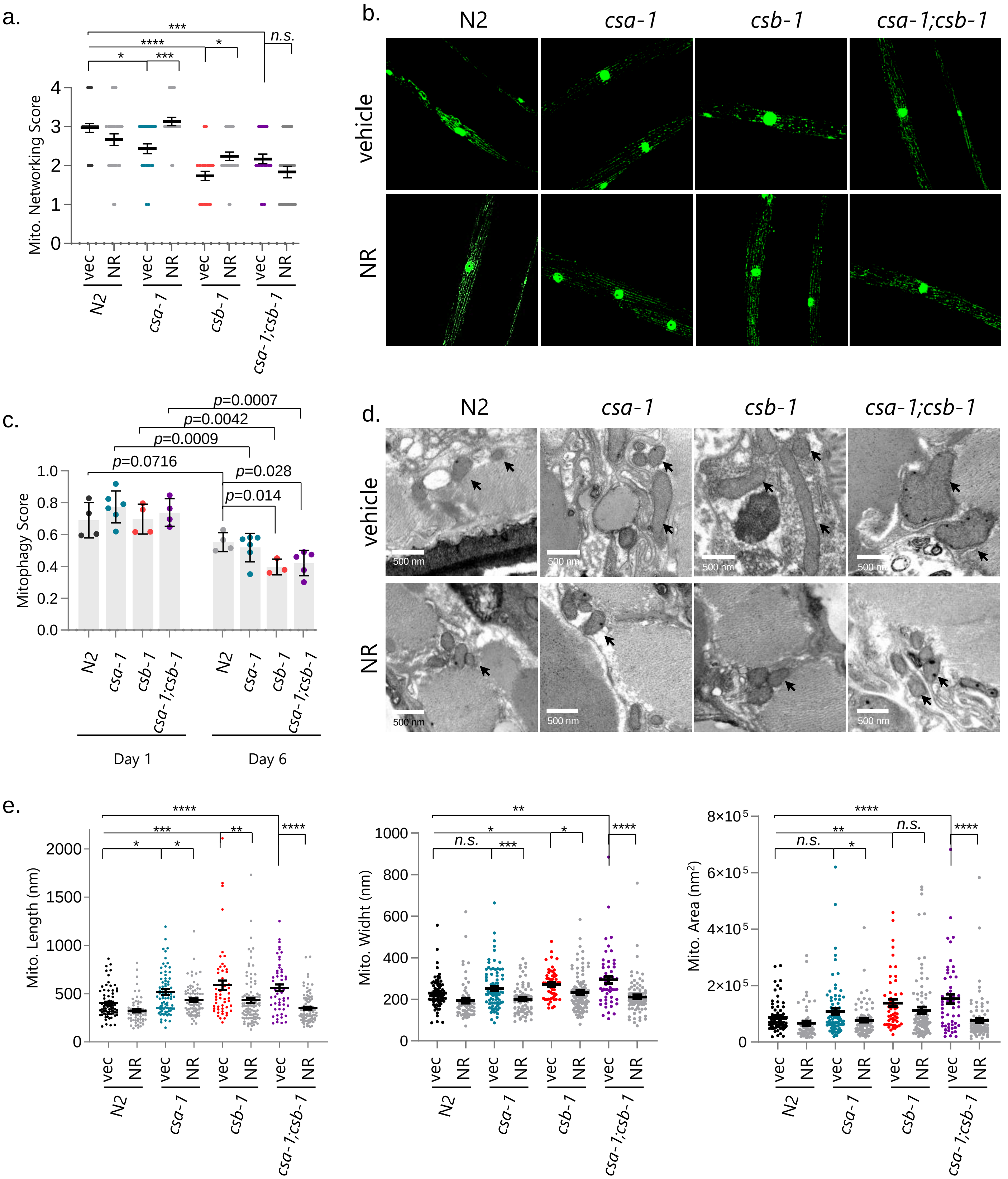

a.
Mito. Networking Score
vec NR vec NR vec NR vec NR
N2
csa-1
csb-1
csa-1;csb-1
n.s.
b.
N2
csa-1
csb-1
csa-1;csb-1
vehicle
NR
c.
Mitophagy Score
p=0.0007
p=0.0042
p=0.0009
p=0.0716
p=0.028
p=0.014
Day 1
Day 6
d.
N2
csa-1
csb-1
csa-1;csb-1
vehicle
NR
500 nm
e.
Mito. Length (nm)
Mito. Widht (nm)
Mito. Area (nm²)

Figure 5

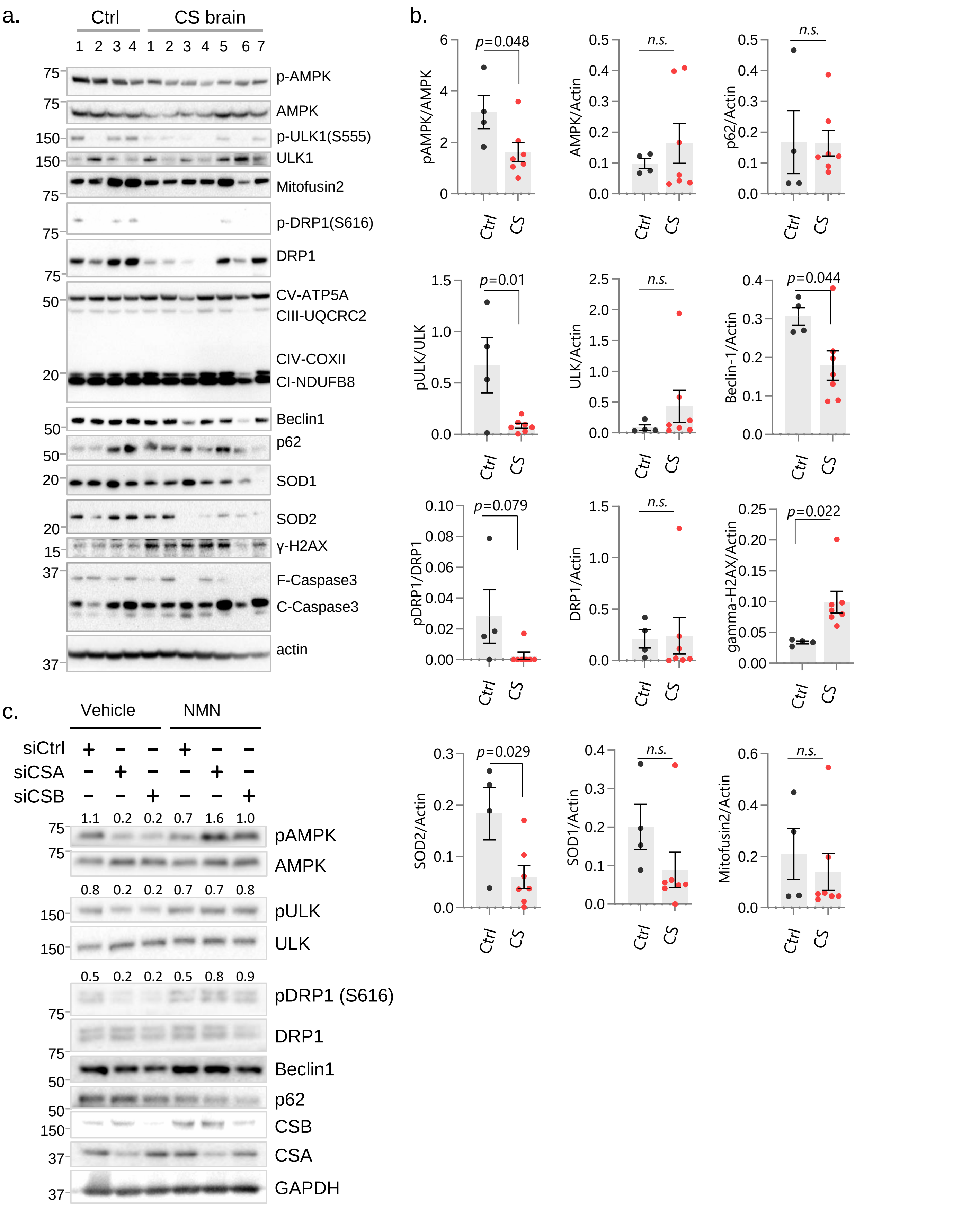

Figure 6

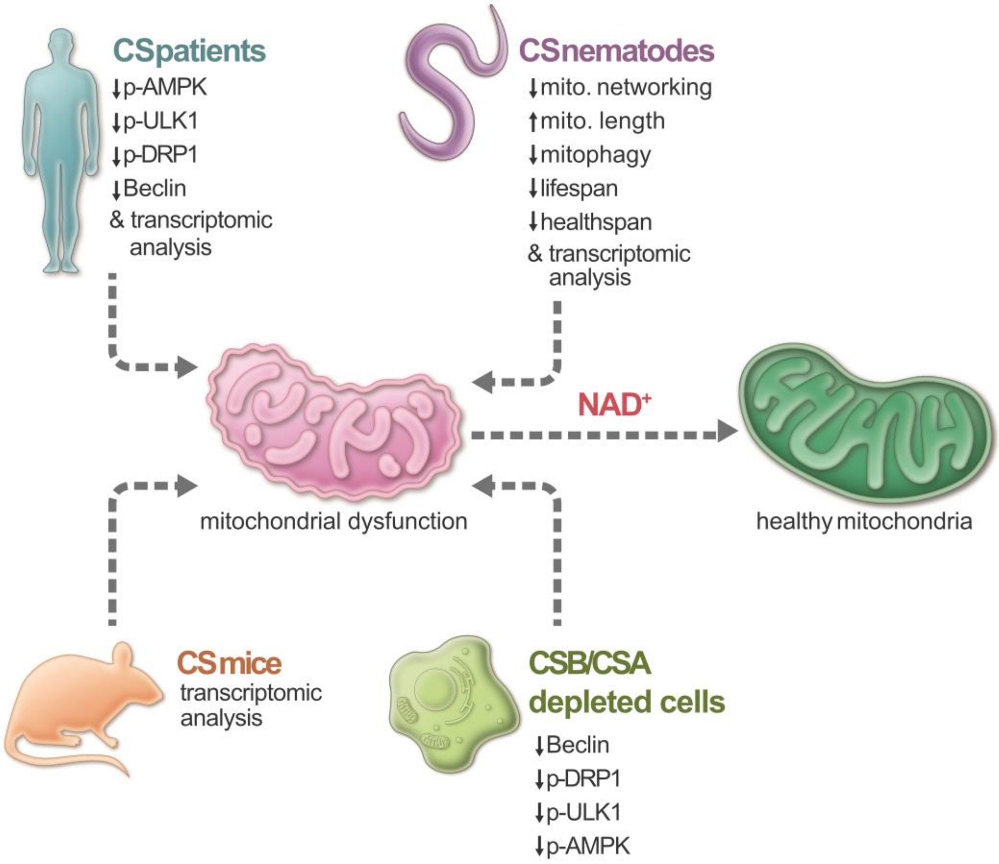

CSpatients
↓p-AMPK
↓p-ULK1
↓p-DRP1
↓Beclin
& transcriptomic analysis
CSnematodes
↓mito. networking
↑mito. length
↓mitophagy
↓lifespan
↓healthspan
& transcriptomic analysis
NAD+
mitochondrial dysfunction
healthy mitochondria
CSmice
transcriptomic analysis
CSB/CSA depleted cells
↓Beclin
↓p-DRP1
↓p-ULK1
↓p-AMPK

Supplementary Figures and Table

Supplementary Table 1

Group	UMBN	Age	Sex	Cause of Death	PMI
Control (WT1)	3415	7	M	Multisystem Failure	12
Control (WT3)	5976	4	F	Smoke Inhalation	21
Control (WT4)	5928	27	F	Cardiac arrhythmia	26
Control (WT5)	1078	17	F	Accident, Multiple Injuries	12
Control (WT6)	5391	8	M	Drowning	12
Cockayne (CS1)	1920	7	M	Diffused alveolar damage / acute pneumonia	16
Cockayne (CS2)	786	17	F	Complication of Disorder	Unknown
Cockayne (CS3)	5492	7	M	Complications of Disorder	Unknown
Cockayne (CS4R)	5581	27	F	Complication of Disorder	14
Cockayne (CS6)	1124	4	F	Complications of Disorder	3
Cockayne (CS7)	5105	8	M	Complication of Disorder	6
Cockayne (CS8)	1762	4	F	Complications of Disorder	8

Supplementary Table 2

Gene Ontology Term (Up-Regulated)	Zscore (Human CS vs Control)
GO0005615 EXTRACELLULAR SPACE	17.1982877
GO0016020 MEMBRANE	14.3827928
GO0006955 IMMUNE RESPONSE	13.89056137
GO0016021 INTEGRAL TO MEMBRANE	13.5362495
GO0046870 CADMIUM ION BINDING	11.90484511
GO0007155 CELL ADHESION	11.54209473
GO0006954 INFLAMMATORY RESPONSE	10.94812908
GO0005737 CYTOPLASM	10.63895048
GO0005520 INSULIN LIKE GROWTH FACTOR BINDING	10.42750915
GO0005739 MITOCHONDRION	10.18613769
GO0005764 LYSOSOME	9.85877125
GO0006956 COMPLEMENT ACTIVATION	9.840334507
GO0045087 INNATE IMMUNE RESPONSE	9.809865555
GO0005576 EXTRACELLULAR REGION	9.735861816
GO0005886 PLASMA MEMBRANE	9.581756988
GO0005578 PROTEINACEOUS EXTRACELLULAR MATRIX	9.360849996
GO0005178 INTEGRIN BINDING	9.303586999
GO0016491 OXIDOREDUCTASE ACTIVITY	9.036500462
GO0005887 INTEGRAL TO PLASMA MEMBRANE	8.849482637
GO0005515 PROTEIN BINDING	8.809728894
GO0019838 GROWTH FACTOR BINDING	8.597717018
GO0006952 DEFENSE RESPONSE	8.500907053
GO0005783 ENDOPLASMIC RETICULUM	8.394284999
GO0004872 RECEPTOR ACTIVITY	8.326364494
GO0005509 CALCIUM ION BINDING	8.298073392
GO0006817 PHOSPHATE TRANSPORT	8.237169775
GO0006935 CHEMOTAXIS	8.127819547
GO0008152 METABOLIC PROCESS	8.083951694
GO0005581 COLLAGEN	7.883409182
GO0005507 COPPER ION BINDING	7.826236993
GO0005604 BASEMENT MEMBRANE	7.745480057
GO0007165 SIGNAL TRANSDUCTION	7.556848936
GO0005198 STRUCTURAL MOLECULE ACTIVITY	7.537305994
GO0030020 EXTRACELLULAR MATRIX STRUCTURAL CONSTITU	7.308711423
GO0004871 SIGNAL TRANSDUCER ACTIVITY	7.174696738
GO0001525 ANGIOGENESIS	7.174292709
GO0016337 CELL CELL ADHESION	7.058800126
GO0007160 CELL MATRIX ADHESION	6.93721382
GO0008284 POSITIVE REGULATION OF CELL PROLIFERATIO	6.904929478
GO0001726 RUFFLE	6.709941735
GO0006928 CELL MOTILITY	6.697710283
GO0007166 CELL SURFACE RECEPTOR LINKED SIGNAL TRAN	6.61859733
GO0003779 ACTIN BINDING	6.522361001
GO0001558 REGULATION OF CELL GROWTH	6.518922008
GO0004364 GLUTATHIONE TRANSFERASE ACTIVITY	6.516375942
GO0006915 APOPTOSIS	6.500390316
GO0001667 AMEBOIDAL CELL MIGRATION	6.377888472
GO0003924 GTPASE ACTIVITY	6.246543791
GO0009615 RESPONSE TO VIRUS	6.235797044
GO0006118 ELECTRON TRANSPORT	6.205399656
GO0005201 EXTRACELLULAR MATRIX STRUCTURAL CONSTITU	6.170621769
GO0008283 CELL PROLIFERATION	6.157249716
GO0003824 CATALYTIC ACTIVITY	6.005319953
GO0006629 LIPID METABOLIC PROCESS	6.002842716
GO0006916 ANTI APOPTOSIS	5.933099193
GO0004180 CARBOXYPEPTIDASE ACTIVITY	5.87220924
GO0005856 CYTOSKELETON	5.861923353
GO0005829 CYTOSOL	5.803244589
GO0015629 ACTIN CYTOSKELETON	5.709100582
GO0008360 REGULATION OF CELL SHAPE	5.706752579
GO0042632 CHOLESTEROL HOMEOSTASIS	5.704322613
GO0005743 MITOCHONDRIAL INNER MEMBRANE	5.69476918
GO0005625 SOLUBLE FRACTION	5.674069526

Gene Ontology Term (Up-Regulated)	Zscore (Human CS vs Control)
GO0030036 ACTIN CYTOSKELETON ORGANIZATION AND BIOG	5.628093311
GO0001569 PATTERNING OF BLOOD VESSELS	5.620061026
GO0008285 NEGATIVE REGULATION OF CELL PROLIFERATIO	5.530971483
GO0007169 TRANSMEMBRANE RECEPTOR PROTEIN TYROSINE	5.498206806
GO0007596 BLOOD COAGULATION	5.447226092
GO0017153 SODIUM DICARBOXYLATE SYMPORTER ACTIVITY	5.391273148
GO0007186 G PROTEIN COUPLED RECEPTOR PROTEIN SIGNA	5.379357197
GO0000074 REGULATION OF PROGRESSION THROUGH CELL C	5.36917598
GO0005506 IRON ION BINDING	5.350705834
GO0005529 SUGAR BINDING	5.345748434
GO0020037 HEME BINDING	5.266458161
GO0007266 RHO PROTEIN SIGNAL TRANSDUCTION	5.239118641
GO0006835 DICARBOXYLIC ACID TRANSPORT	5.082855262
GO0030301 CHOLESTEROL TRANSPORT	5.065979341
GO0009897 EXTERNAL SIDE OF PLASMA MEMBRANE	4.970319849
GO0000302 RESPONSE TO REACTIVE OXYGEN SPECIES	4.958212236
GO0046983 PROTEIN DIMERIZATION ACTIVITY	4.947945581
GO0005540 HYALURONIC ACID BINDING	4.868653021
GO0005096 GTPASE ACTIVATOR ACTIVITY	4.83334221
GO0007267 CELL CELL SIGNALING	4.674093335
GO0004888 TRANSMEMBRANE RECEPTOR ACTIVITY	4.668652335
GO0008305 INTEGRIN COMPLEX	4.60079215
GO0006950 RESPONSE TO STRESS	4.525745881
GO0043123 POSITIVE REGULATION OF I KAPPAB KINASE O	4.490558395
GO0005792 MICROSOME	4.45435458
GO0042803 PROTEIN HOMODIMERIZATION ACTIVITY	4.373368183
GO0005125 CYTOKINE ACTIVITY	4.365701952
GO0048469 CELL MATURATION	4.358338135
GO0016740 TRANSFERASE ACTIVITY	4.356555507
GO0043169 CATION BINDING	4.288798199
GO0008610 LIPID BIOSYNTHETIC PROCESS	4.275246694
GO0005794 GOLGI APPARATUS	4.220300217
GO0001584 RHODOPSIN LIKE RECEPTOR ACTIVITY	4.21577683
GO0006508 PROTEOLYSIS	4.210195997
GO0006826 IRON ION TRANSPORT	4.209012135
GO0016787 HYDROLASE ACTIVITY	4.208267494
GO0005975 CARBOHYDRATE METABOLIC PROCESS	4.169760623
GO0042098 T CELL PROLIFERATION	4.150633129
GO0004930 G PROTEIN COUPLED RECEPTOR ACTIVITY	4.135672599
GO0008233 PEPTIDASE ACTIVITY	4.10297131
GO0005626 INSOLUBLE FRACTION	4.083811492
GO0005172 VASCULAR ENDOTHELIAL GROWTH FACTOR RECEP	4.030144773
GO0043065 POSITIVE REGULATION OF APOPTOSIS	4.026445742
GO0005777 PEROXISOME	3.89659395
GO0005575 CELLULAR COMPONENT	3.731950662
GO0016126 STEROL BIOSYNTHETIC PROCESS	3.729420062
GO0042981 REGULATION OF APOPTOSIS	3.691246454
GO0003674 MOLECULAR FUNCTION	3.658278631
GO0008415 ACYLTRANSFERASE ACTIVITY	3.590434743
GO0008652 AMINO ACID BIOSYNTHETIC PROCESS	3.580936711
GO0009987 CELLULAR PROCESS	3.516975886
GO0006357 REGULATION OF TRANSCRIPTION FROM RNA POL	3.477296205
GO0016599 CAVEOLAR MEMBRANE	3.433956615
GO0005622 INTRACELLULAR	3.305133507
GO0006635 FATTY ACID BETA OXIDATION	3.288547101
GO0005901 CAVEOLA	3.248459924
GO0051607 DEFENSE RESPONSE TO VIRUS	3.157754094
GO0030203 GLYCOSAMINOGLYCAN METABOLIC PROCESS	3.006477472
GO0030111 REGULATION OF WNT RECEPTOR SIGNALING PAT	2.897879222
GO0005667 TRANSCRIPTION FACTOR COMPLEX	2.8870436
GO0006939 SMOOTH MUSCLE CONTRACTION	2.83018972
GO0005761 MITOCHONDRIAL RIBOSOME	2.566761664
GO0005762 MITOCHONDRIAL LARGE RIBOSOMAL SUBUNIT	2.303794977

Gene Ontology Term (Down-Regulated)	Zscore (Human CS vs Control)
GO0045202 SYNAPSE	-7.814045563
GO0007269 NEUROTRANSMITTER SECRETION	-5.699196157
GO0007268 SYNAPTIC TRANSMISSION	-4.617110766
GO0030672 SYNAPTIC VESICLE MEMBRANE	-4.589458601
GO0017157 REGULATION OF EXOCYTOSIS	-4.388510477
GO0045211 POSTSYNAPTIC MEMBRANE	-4.221094657
GO0007411 AXON GUIDANCE	-4.21386283
GO0043005 NEURON PROJECTION	-3.798158591
GO0006512 UBIQUITIN CYCLE	-3.508568512
GO0006376 MRNA SPLICE SITE SELECTION	-3.188734945
GO0004221 UBIQUITIN THIOLESTERASE ACTIVITY	-3.164510276
GO0016568 CHROMATIN MODIFICATION	-2.971789514
GO0007270 NERVE NERVE SYNAPTIC TRANSMISSION	-2.802517129
GO0005832 CHAPERONIN CONTAINING T COMPLEX	-2.589379785
GO0016363 NUCLEAR MATRIX	-2.370025075
GO0035097 HISTONE METHYLTRANSFERASE COMPLEX	-2.186841552
GO0006754 ATP BIOSYNTHETIC PROCESS	-2.052757727

Supplementary Table 3

	UMBN	Age	Sex	Cause of death	PMI
#Ctrl-1	5976	4	Female	Smoke Inhalation	21
#Ctrl-2	5928	27	Female	Cardiac arrythmia	26
#Ctrl-3	1078	17	Female	Accident, Multiple Injuries	12
#Ctrl-4	5391	8	Male	Drowning	12
#CS-1	1920	7	Male	Diffused alveolar damage / acute pneumonia	16
#CS-2	786	17	female	Complications of Disorder	22
#CS-3	5492	7	Male	Complications of Disorder	unknown
#CS-4	1286	5	Male	Complications of Disorder	14
#CS-5	1124	4	Female	Complications of Disorder	3
#CS-6	5105	8	Male	Complications of Disorder	6
#CS-7	1762	4	Female	Complications of Disorder	8

Supplementary Figure 1

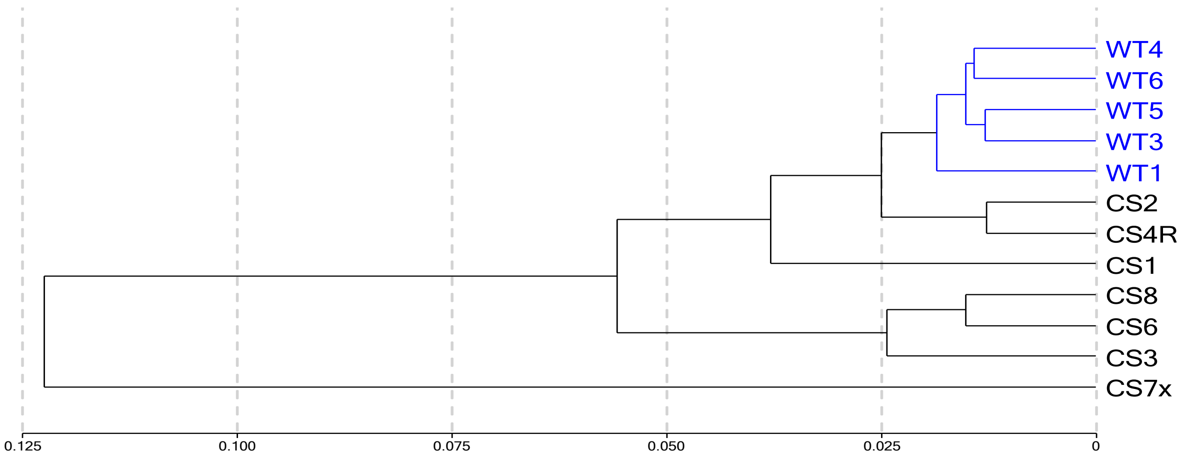

Supplementary Figure 2

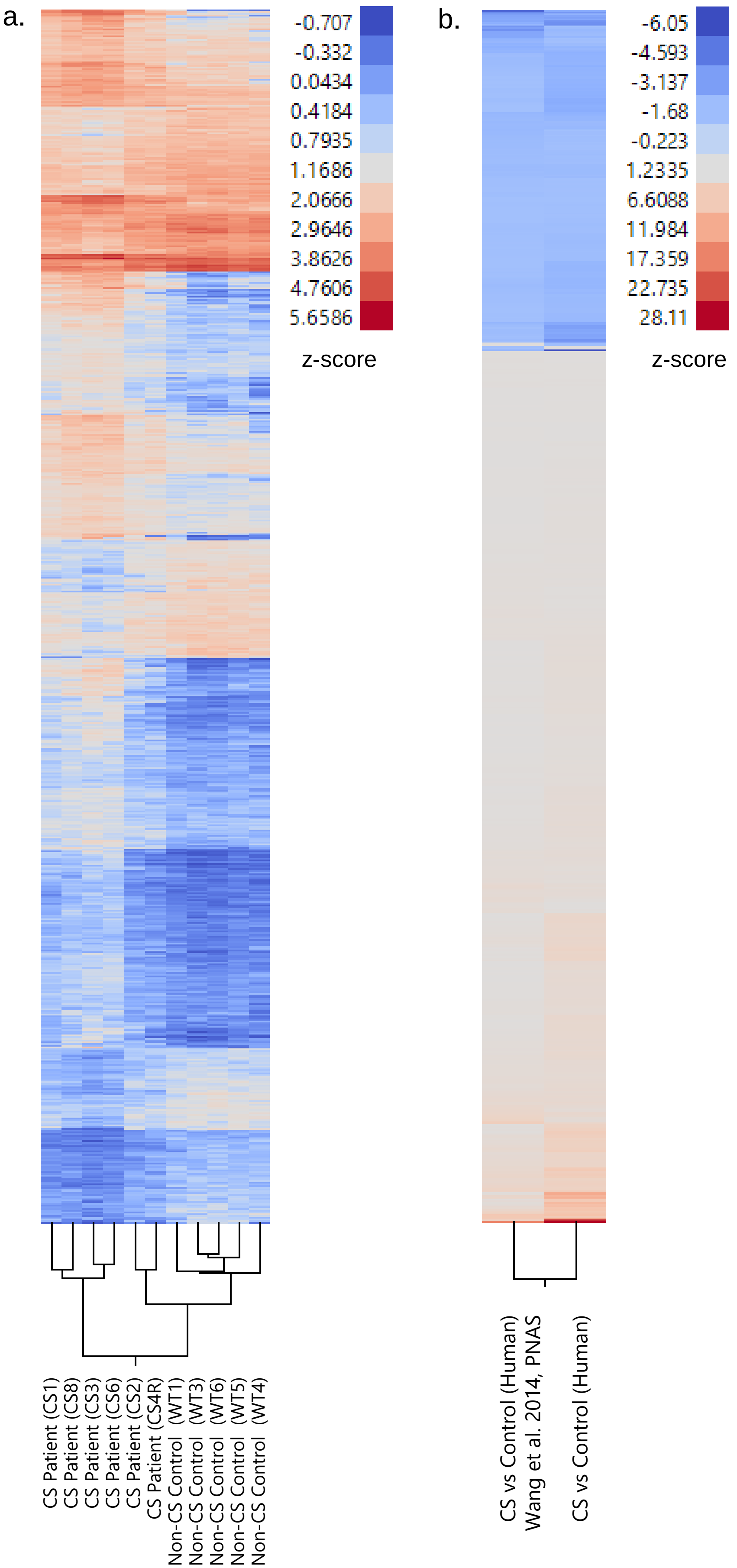

Supplementary Figure 3

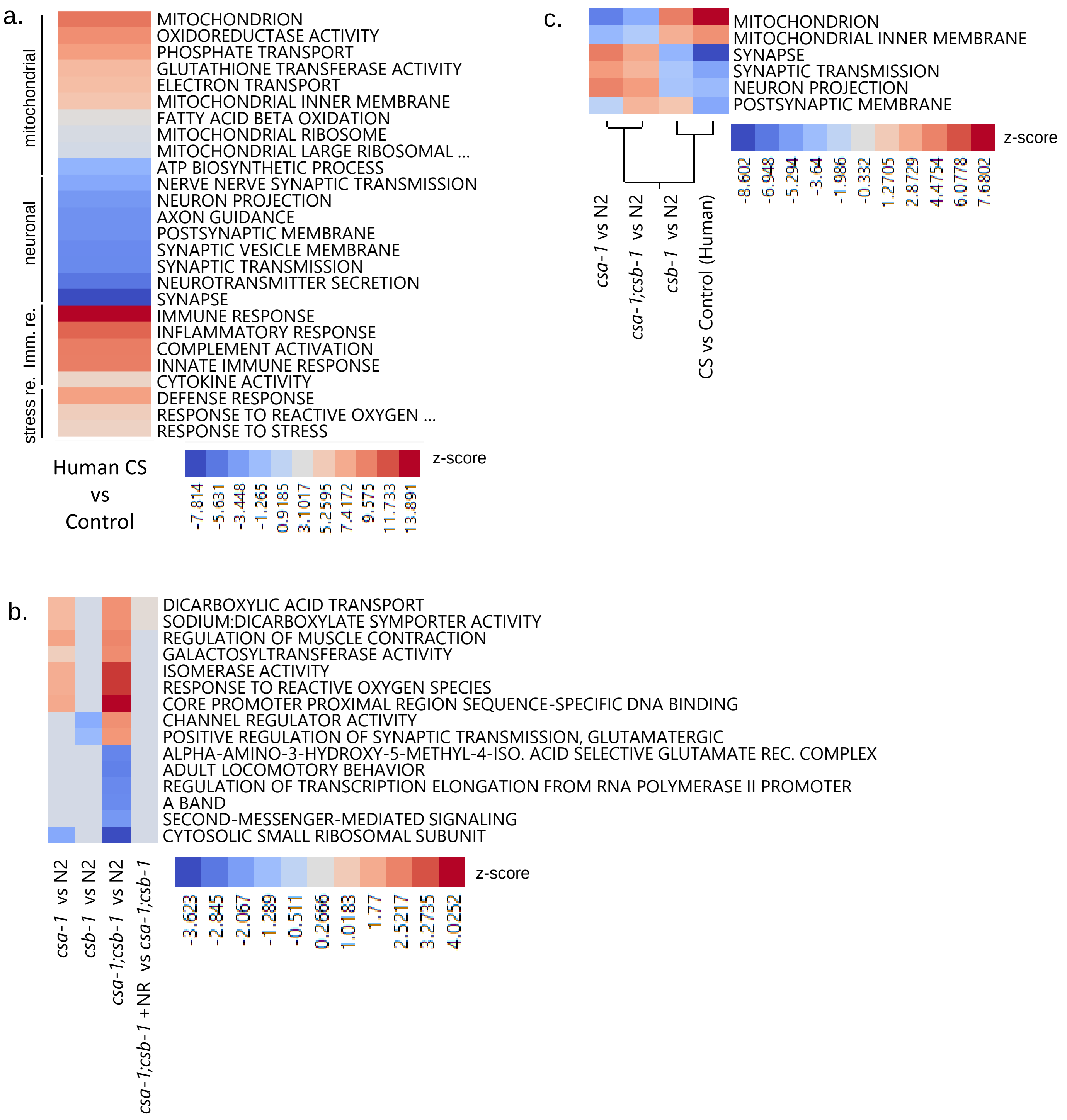

Supplementary Figure 4

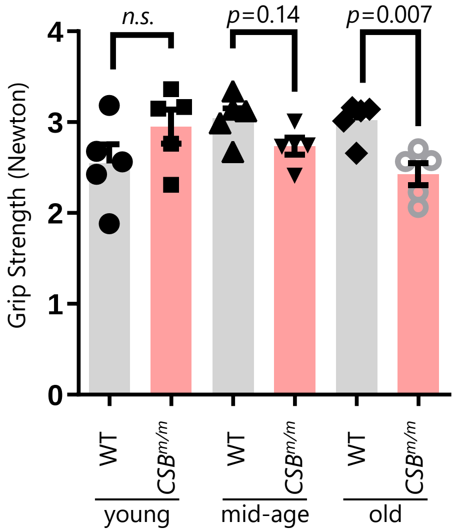

Supplementary Figure 5

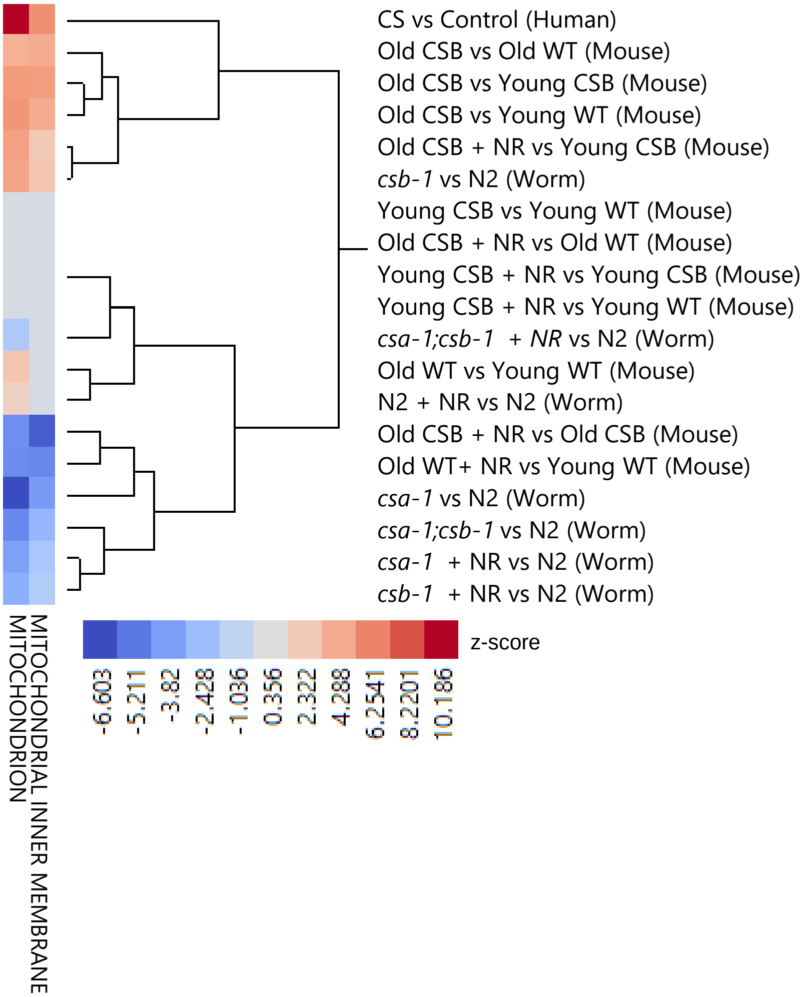

Supplementary Figure 6

a.

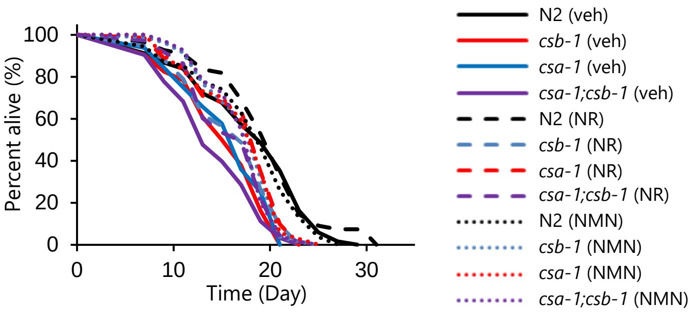

b.

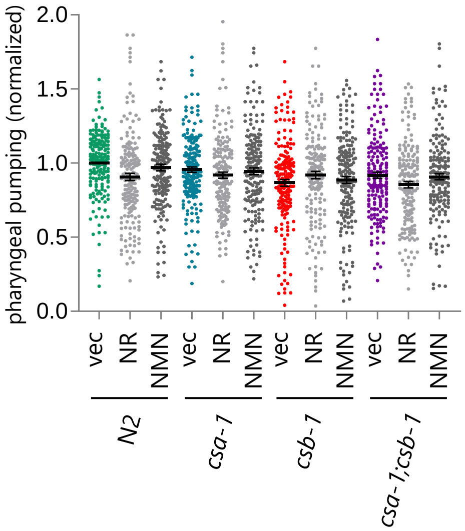

c.

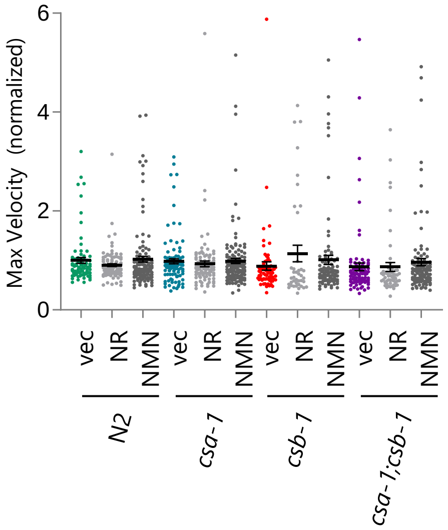

Supplementary Figure 7

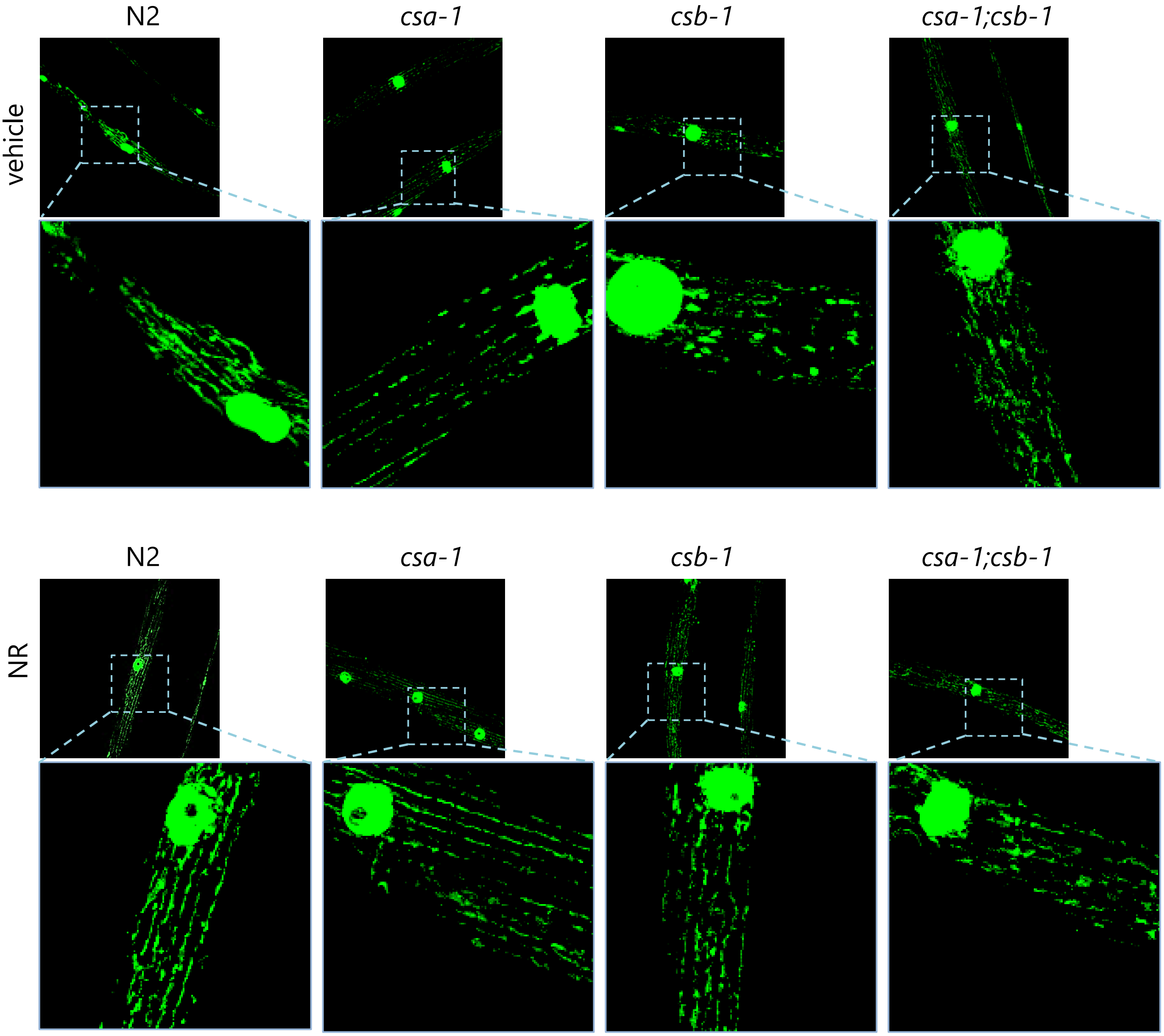

Supplementary Figure 8

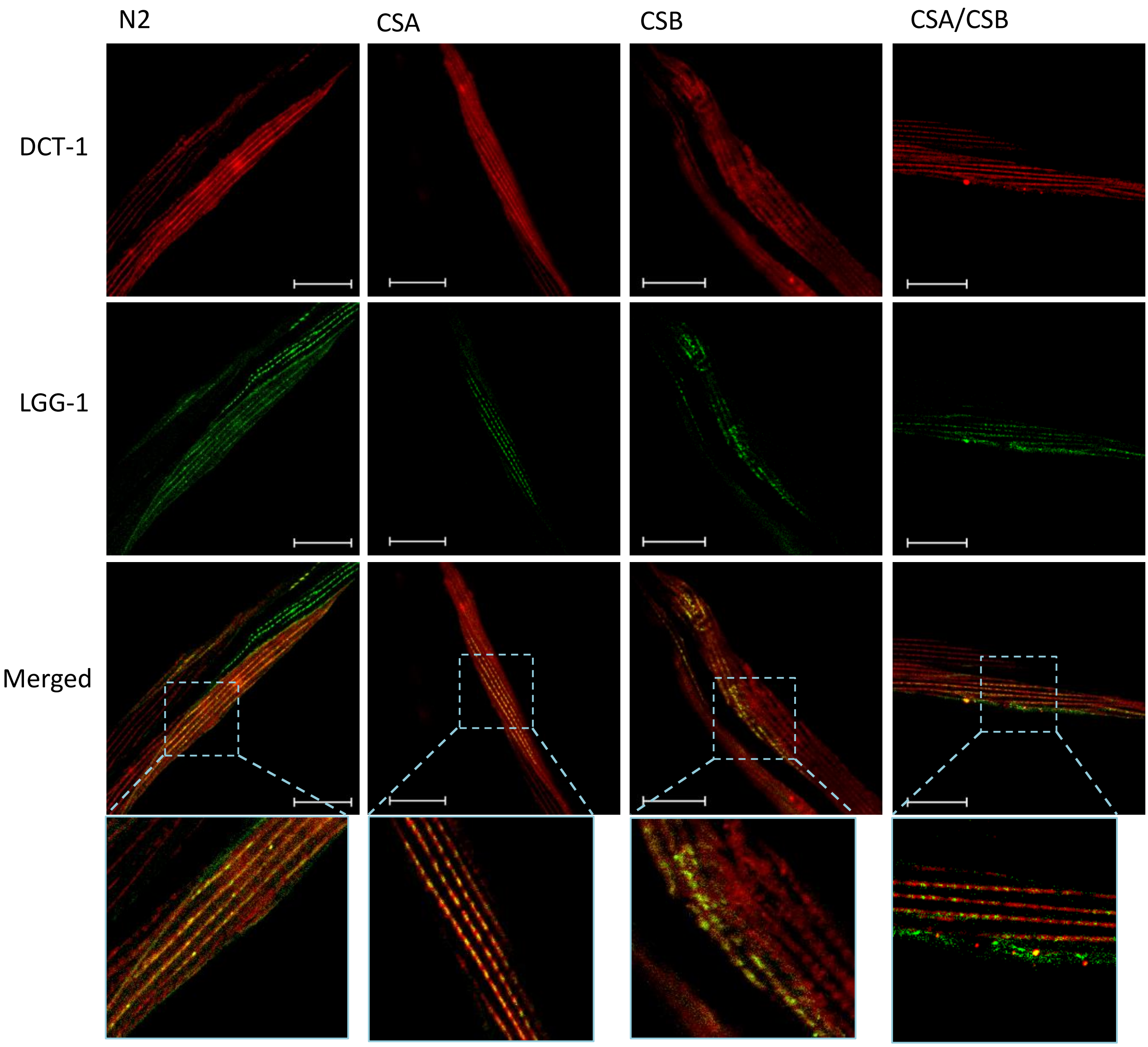

Supplementary Figure 9

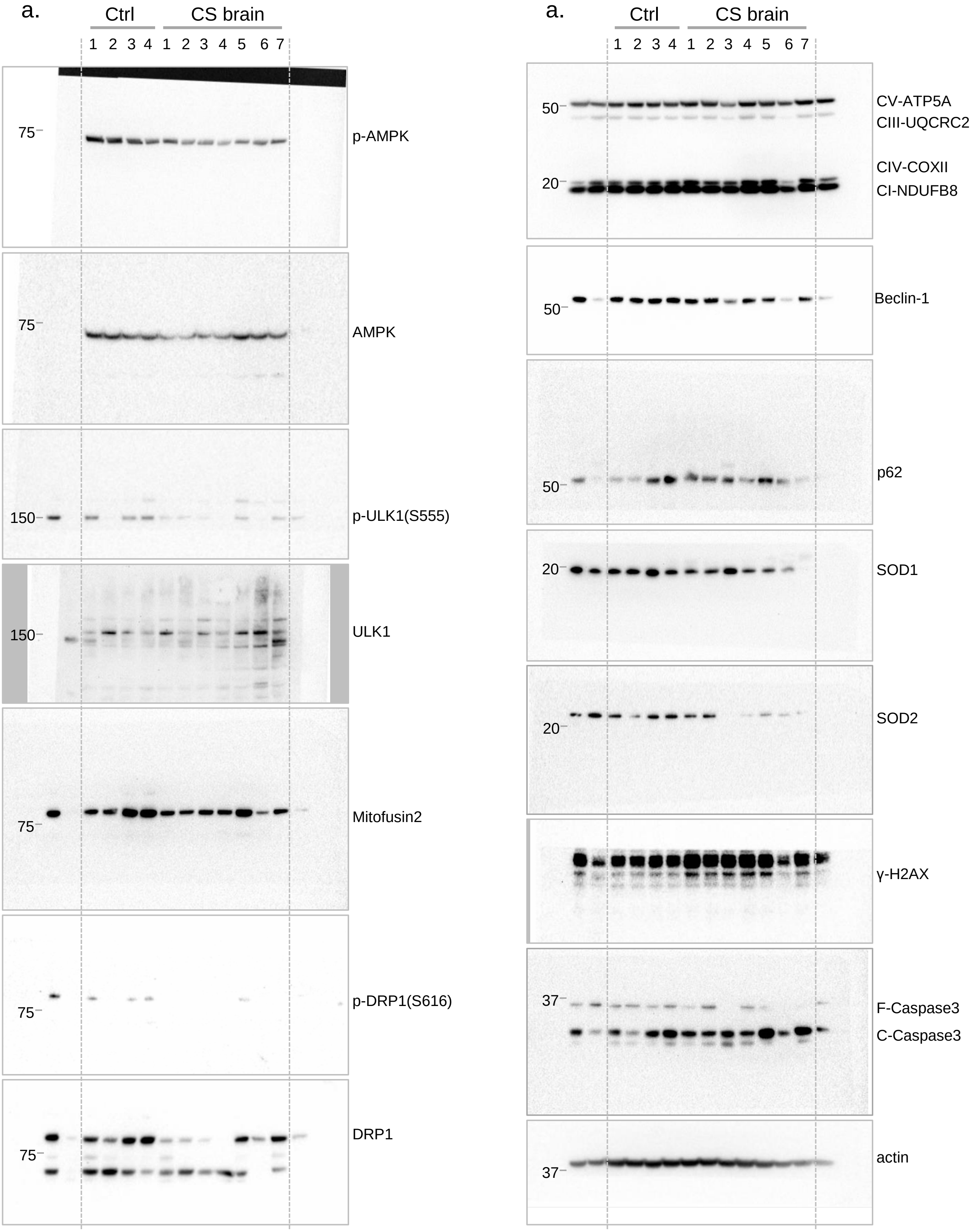

Supplementary Figure 10

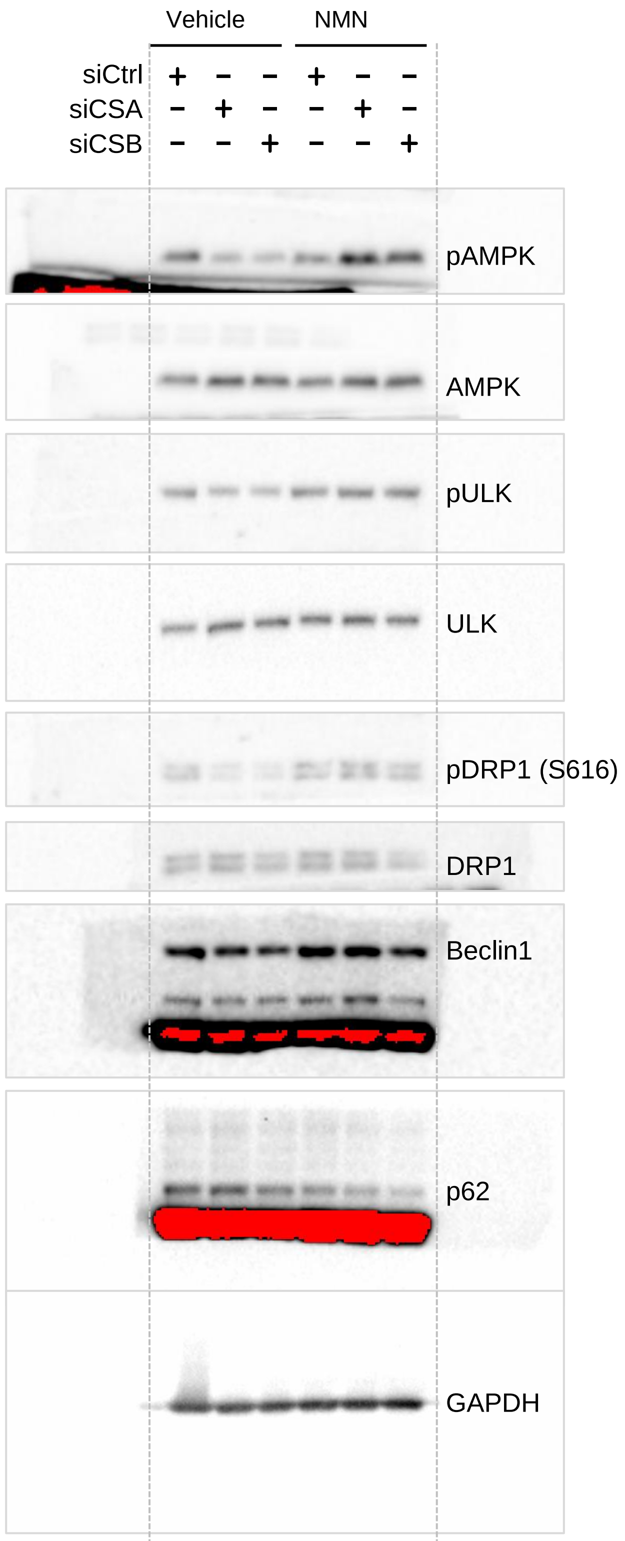

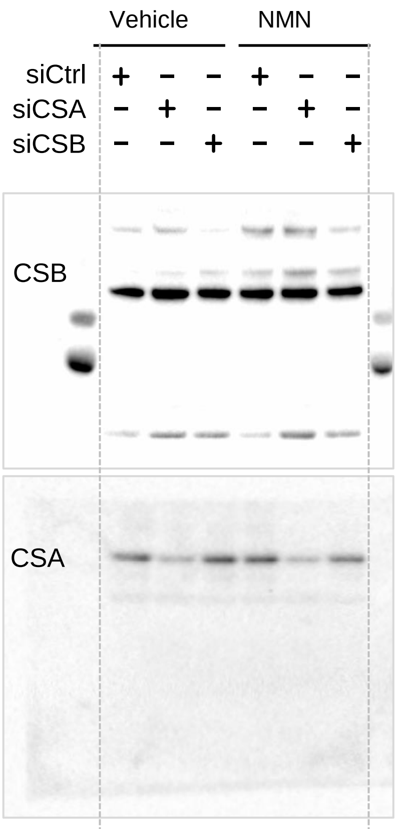

Supplementary Figure 11

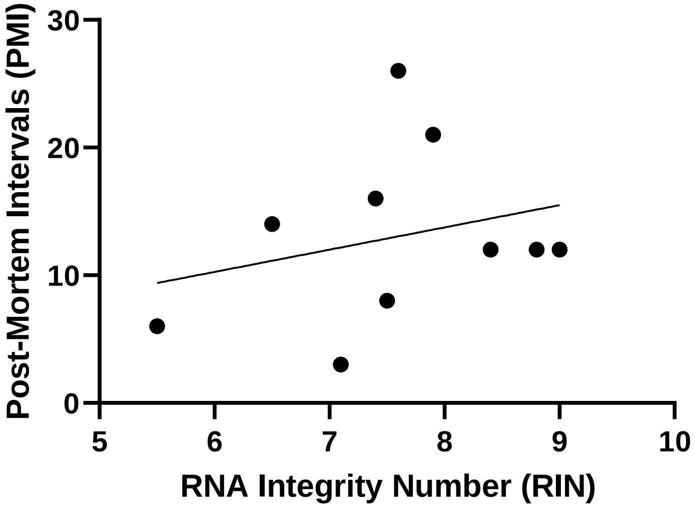

Pearson r	
r	0.2695
95% confidence interval	-0.4337 to 0.7687
R squared	0.07262
